# The crystal structures of salts of *N*-(4-fluoro­phen­yl)piperazine with four aromatic carb­oxy­lic acids and with picric acid

**DOI:** 10.1107/S2056989020008749

**Published:** 2020-07-03

**Authors:** Chayanna Harish Chinthal, Hemmige S. Yathirajan, Channappa N. Kavitha, Sabine Foro, Christopher Glidewell

**Affiliations:** a Department of Studies in Chemistry, University of Mysore, Manasagangotri, Mysuru-570 006, India; b Department of Chemistry, Maharani’s Science College for Women, Mysuru-570 001, India; c Institute of Materials Science, Darmstadt University of Technology, Alarich-Weiss-Strasse 2, D-64287 Darmstadt, Germany; d School of Chemistry, University of St Andrews, St Andrews, Fife KY16 9ST, UK

**Keywords:** piperazines, piperazine salts, crystal structure, mol­ecular structure, hydrogen bonding, supra­molecular assembly

## Abstract

The structures are reported for five salts formed by reactions between *N*-(4-fluoro­phen­yl)piperazine and aromatic acids. In 4-(4-fluoro­phen­yl)piperazin-1-ium 2-fluoro­benzoate monohydrate, C_10_H_14_FN_2_^+^·C_7_H_4_FO_2_^−^·H_2_O, (I), the components are linked by a combination of N—H⋯O and O—H⋯O hydrogen bonds to form a chain of alternating *R* ^4^_6_(12) and *R* ^6^_6_(16) rings. The ionic components of 4-(4-fluoro­phen­yl)piperazin-1-ium 2-bromo­benzoate 0.353-hydrate, C_10_H_14_FN_2_^+^·C_7_H_4_BrO_2_^−^·0.353H_2_O, (II), are linked by N—H⋯O hydrogen bonds to form a centrosymmetric four-ion aggregate containing an *R* ^4^_4_(12) motif, and these aggregates are linked into a mol­ecular ladder by a single C—H⋯π(arene) hydrogen bond. 4-(4-Fluoro­phen­yl)piperazin-1-ium 2-iodo­benzoate, C_10_H_14_FN_2_^+^·C_7_H_4_IO_2_^−^, (III), crystallizes with *Z*′ = 2 in space group *P* : the four independent ions are linked by N—H⋯O hydrogen bonds to form a non-centrosymmetric aggregate again containing an *R* ^4^_4_(12) motif, and aggregates of this type are linked into a ribbon by a combination of C—H⋯O and C—H⋯π(arene) hydrogen bonds. The anion in 4-(4-fluoro­phen­yl)piperazin-1-ium 2,4,6-tri­nitro­phenolate, C_10_H_14_FN_2_^+^·C_6_H_2_N_3_O_7_^−^, (IV), shows clear evidence of extensive electronic delocalization from the phenolate O atom into the adjacent ring. The ions are linked by a combination of two-centre N—H⋯O and three-centre N—H⋯(O)_2_ hydrogen bonds to form centrosymmetric four-ion aggregates containing three types of ring. The ions in 4-(4-fluoro­phen­yl)piperazin-1-ium 3,5-di­nitro­benzoate, C_10_H_14_FN_2_^+^·C_7_H_3_N_2_O_6_^−^, (V), are again linked by N—H⋯O hydrogen bonds to form centrosymmetric *R* ^4^_4_(12) aggregates, which are themselves linked by a C—H⋯π(arene) hydrogen bond to form sheets, the stacking of which leads to the formation of narrow channels, containing disordered and/or mobile solvent entities. Comparisons are made with some related structures.

## Abstract 2

The structures of five 4-(4-fluoro­phen­yl)piperazin-1-ium salts of closely related aromatic acids all show different patterns of supra­molecular assembly.

## Chemical context   

*N*-(4-fluoro­phen­yl)piperazine (C_10_H_13_N_2_F; 4-FPP) has mild psychedelic and euphorigenic properties and, in this respect, it exhibits effects similar to those of the related compound *N*-(4-meth­oxy­phen­yl)piperazine (MeOPP), which has been used as a recreational drug (Nagai *et al.*, 2007 ▸). 4-FPP is also is a major metabolite (Keane *et al.*, 1982 ▸; Sanjuan *et al.*, 1983 ▸) of the sedative and hypnotic drug niaprazine, *N*-{4-[4-(4-fluoro­phen­yl)piperazin-1-yl]butan-2-yl}pyridine-3-carboxamide, used in the treatment of autistic disorders (Rossi *et al.*, 1999 ▸).

**Figure 1:**
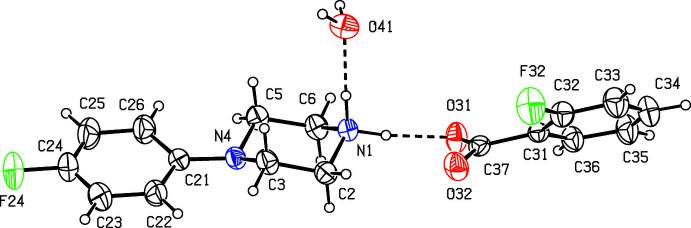
The independent components of compound (I) showing the atom-labeling scheme and the hydrogen bonds, drawn as dashed lines, within the selected asymmetric unit. Displacement ellipsoids are drawn at the 30% probability level.

We have recently reported (Harish Chinthal *et al.*, 2020 ▸) the structures of the salts formed between 4-FPP and 2-hy­droxy-3,5-di­nitro­benzoic, oxalic and (2*R*,3*R*)-tartaric acids, the last of which crystallizes as a monohydrate. That work was a development from our structural studies (Kiran Kumar *et al.*, 2019 ▸, 2020 ▸) of a wide range of salts formed between organic acids and MeOPP. As part of our study of 4-FPP, we now report the structures of five salts formed between 4-FPP and four aromatic carb­oxy­lic acids and picric acid, namely 4-(4-fluoro­phen­yl)piperazin-1-ium 2-fluoro­benzoate monohydrate (I), 4-(4-fluoro­phen­yl)piperazin-1-ium 2-bromo­benzoate 0.353(hydrate) (II), 4-(4-fluoro­phen­yl)piperazin-1-ium 2-iodo­benzoate (III), 4-(4-fluoro­phen­yl)piperazin-1-ium 2,4,6-tri­nitro­phenolate (IV) and 4-(4-fluoro­phen­yl)piperazin-1-ium 3,5-di­nitro­benzoate (V).[Chem scheme1] (Figs. 1 ▸–5 ▸ ▸ ▸ ▸)
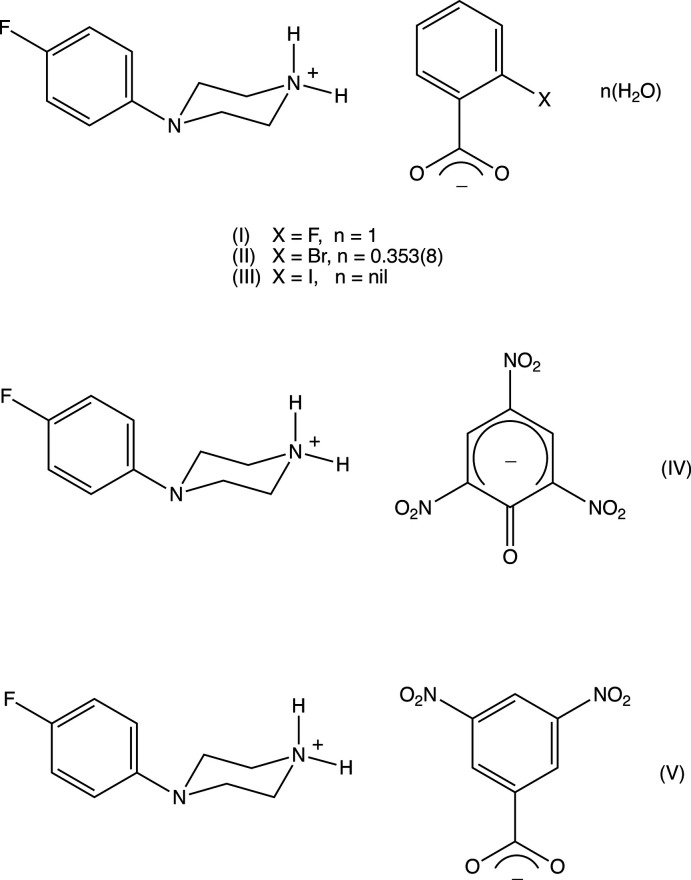


## Structural commentary   

**Figure 2:**
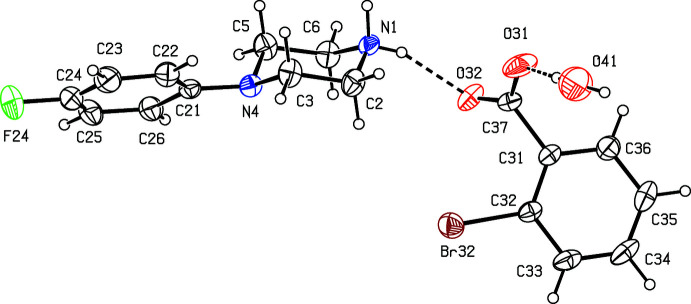
The independent components of compound (II) showing the atom-labeling scheme and the hydrogen bonds, drawn as dashed lines, within the selected asymmetric unit. The water mol­ecule has occupancy 0.353 (8) and the displacement ellipsoids are drawn at the 30% probability level.

**Figure 3:**
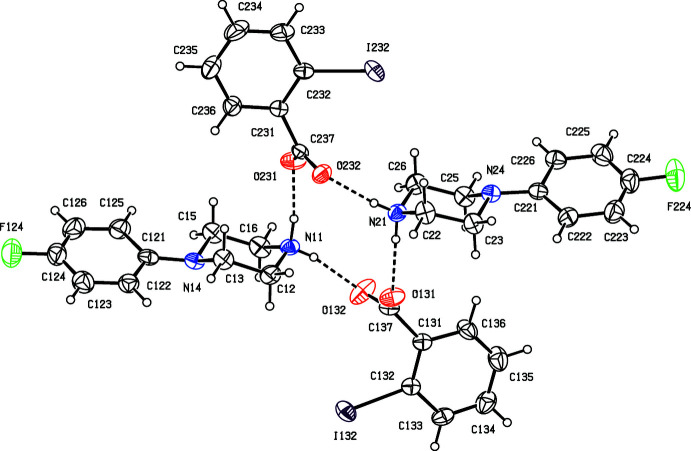
The independent components of compound (III) showing the atom-labeling scheme and the hydrogen bonds, drawn as dashed lines, within the selected asymmetric unit. Displacement ellipsoids are drawn at the 30% probability level.

The crystallization characteristics of the 2-halobenzoate salts (I)–(III) are all different (Figs. 1 ▸–3 ▸ ▸), so that no two of them are isostructural. The 2-fluoro­benzoate salt (I) crystallizes as a monohydrate. However, the 2-bromo­benzoate (II) crystallizes as a partial hydrate: the refined occupancy of the water mol­ecule is 0.353 (8) and the O atom of this component, O41 (Fig. 2 ▸), lies close to an inversion centre, such that the O⋯O distance across this centre is only 0.962 (16) Å. Hence, if either of this pair of sites is occupied, the other must be vacant. By contrast, the 2-iodo­benzoate (III) crystallizes in solvent-free form with Z′ = 2 in space group *P* 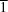 (Fig. 3 ▸). The refined structure of the 3,5-di­nitro­benzoate salt (V) contains four void spaces per unit cell, each of volume 59 Å^3^, which lie on the twofold rotation axes, and which are connected into narrow channels lying along these axes. Examination of the refined structure using the SQUEEZE procedure (Spek, 2015 ▸) showed the the presence of 48 electrons per unit cell that were not accounted for by the ionic components, *i.e*. an average of 12 electrons per void, or rather less than the equivalent of one water mol­ecule. There were two significant peaks in the difference maps, but no plausible solvent model could be developed from these. Hence the SQUEEZE procedure was used prior to the final refinement, and the nature of the solvent component remains unknown: it seem likely that the included mol­ecules are disordered and/or mobile within the channels.

In each of the cations in compounds (I)–(V), the piperazine ring adopts an almost perfect chair conformation: in every case the reference cation was selected as one having the ring-puckering angle θ (Cremer & Pople, 1975 ▸), as calculated for the atom sequence (N1,C2,C3,N4,C5,C6), or the equivalent sequences in compound (III), close to the ideal value (Boeyens, 1978 ▸) of zero, rather than close to 180° as expected for the enanti­omeric form of the cation.

The dihedral angles between the aryl ring and the carboxyl­ate group in compounds (I)–(III) and (V) vary from 5.94 (11)° in (V) to 75.9 (2)° in the anion of (III), which contains atom I132 (Fig. 3 ▸). The corresponding angles involving the nitro groups in compounds (IV) and (V) span a much smaller range, from 5.24 (11)° for the group containing atom N35 in (V) to 29.09 (6)° for the group containing atom N36 in (IV). This contrasting behaviour may be associated with the differences in the hydrogen-bonding participation of the carboxyl­ate and nitro groups, as discussed below (§ 3).

Within the anion of compound (IV), the distances C31—C32 and C31—C36 of 1.451 (2) and 1.449 (2) Å, respectively are very much longer than the other C—C distances in this ring, which range from 1.365 (2) Å to 1.383 (2) Å; in addition the distance C31—O31 [1.2398 (19) Å] is much close to the values typically found in ketones than to those in phenols (Allen *et al.*, 1987 ▸). These values indicate significant delocalization of the negative charge from the atom O31 into the adjacent ring, as shown in the Scheme.

## Supra­molecular features   

It is possible to select a compact asymmetric unit for compound (I) (Fig. 1 ▸) in which the three independent components are linked by two N—H⋯O hydrogen bonds (Table 1 ▸). The supra­molecular assembly of (I) is determined by a combination of two N—H⋯O hydrogen bonds and two O—H⋯O hydrogen bonds (Table 1 ▸). Together these link the three independent components into a chain of centrosymmetric rings running parallel to the [100] direction in which rings of 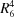
(12) type (Bernstein *et al.*, 1995 ▸) centred at (*n*, 0.5, 0.5) alternate with 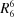
(16) rings centred at (*n* + 0.5, 0.5, 0.5), where *n* represents an integer in each case (Fig. 6 ▸). A weak C—H⋯O hydrogen bond (Table 1 ▸), having a fairly small C—H⋯O angle (Wood *et al.*, 2009 ▸), links these chains into complex sheets lying parallel to (001).

The ionic components of compound (II) are linked by two independent N—H⋯O hydrogen bonds (Table 1 ▸) to form a centrosymmetric four-ion aggregate, characterized by an 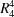
(12) motif, with the reference aggregate centred at (0.5, 0.5, 0.5) (Fig. 7 ▸). If the water mol­ecules were present with full occupancy, their role would be the linking of the four-ion aggregates into chains running parallel to the [010] direction: however, the low occupancy of the water sites indicates that continuous chain formation is not possible. On the other hand, a single C—H⋯π(arene) hydrogen bond links these four-ion aggregates into a ribbon, or mol­ecular ladder running parallel to the [100] direction (Fig. 8 ▸), in which the centrosymmetric 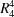
(12) rings containing four N—H⋯O hydrogen bonds and centred at (*n* + 0.5, 0.5, 0.5) alternate with rings containing two each of N—H⋯O and C—H⋯π(arene) hydrogen bonds and centred at (*n*, 0.5, 0.5), where *n* represents an integer in each case.

As noted above, compound (III) crystallizes with *Z*′ = 2 (Fig. 3 ▸) and it is possible to select a compact asymmetric unit in which the four independent ions are linked by four N—H⋯O hydrogen bonds to form a four-ion 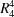
(12) aggregate, analogous to that in compound (II). The aggregate in (III) exhibits approximate, but non-crystallographic, inversion symmetry with its centroid close to (0.25, 0.25, 0.5): a search for possible additional crystallographic symmetry found none. A combination of one C—H⋯O and one C—H⋯π(arene) hydrogen bonds (Table 1 ▸) links the four-ion aggregates into a complex mol­ecular ribbon running parallel to the [010] direction (Fig. 9 ▸).

**Figure 4:**
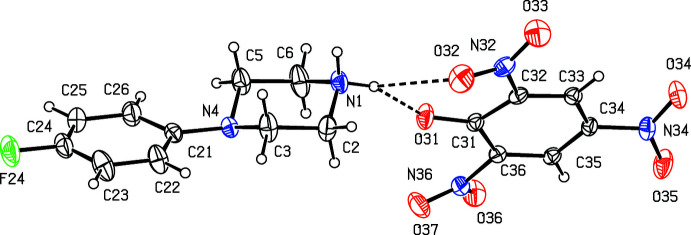
The independent components of compound (IV) showing the atom-labeling scheme and the hydrogen bonds, drawn as dashed lines, within the selected asymmetric unit. Displacement ellipsoids are drawn at the 30% probability level.

**Figure 6:**
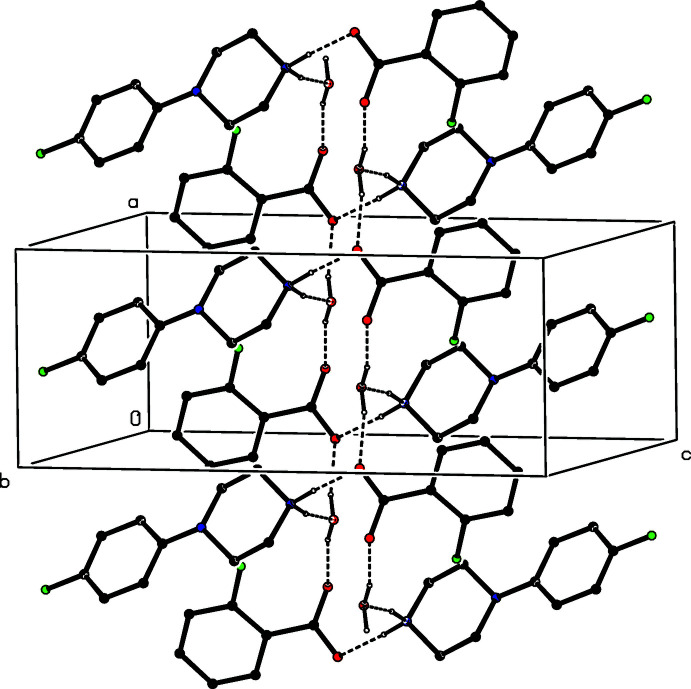
Part of the crystal structure of compound (I) showing the formation of a hydrogen-bonded chain of alternating 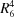
(12) and 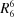
(16) rings along [100]. Hydrogen bonds are drawn as dashed lines and, for the sake of clarity, the H atoms bonded to C atoms have been omitted.

**Figure 7:**
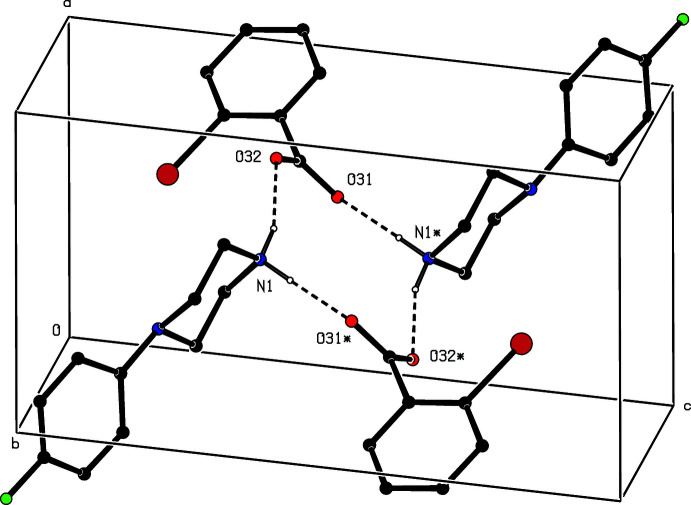
Part of the crystal structure of compound (II) showing the formation of a centrosymmetric four-ion 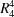
(12) aggregate. Hydrogen bonds are drawn as dashed lines and, for the sake of clarity, the partial-occupancy water mol­ecules and the H atoms bonded to C atoms have been omitted. The atoms marked with an asterisk (*) are at the symmetry position (1 − *x*, 1 − *y*, 1 − *z*).

**Figure 8:**
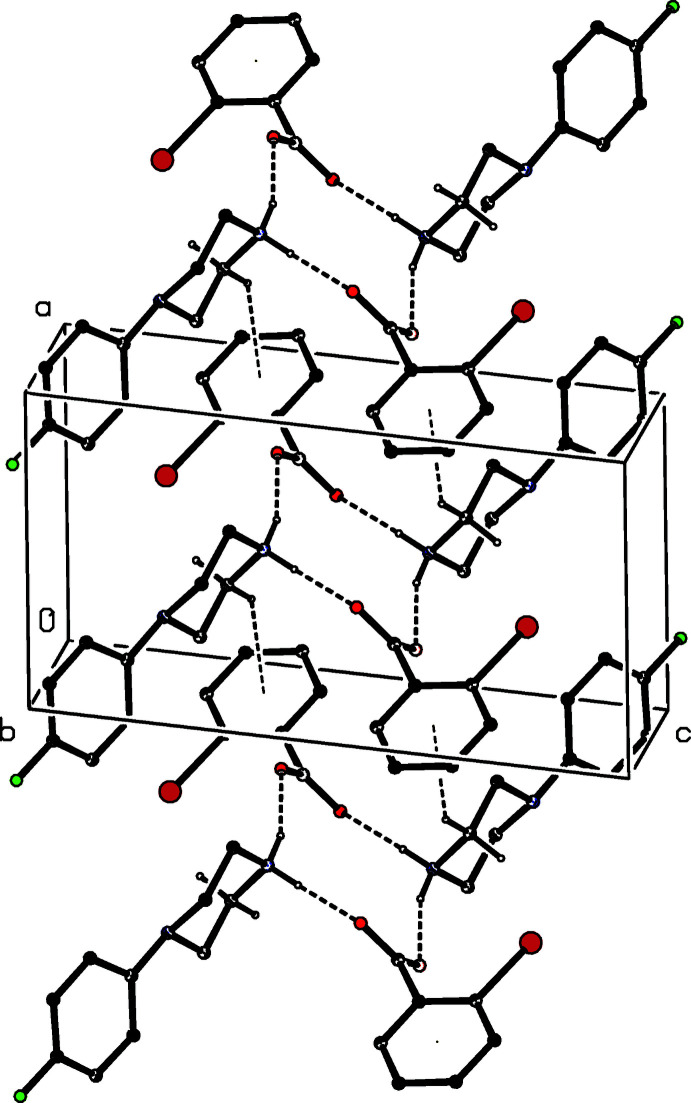
Part of the crystal structure of compound (II) showing the formation of a mol­ecular ribbon of centrosymmetric rings running parallel to the [100] direction. Hydrogen bonds are drawn as dashed lines and, for the sake of clarity, the partial-occupancy water mol­ecules and the H atoms bonded to those C atoms which are not involved in the motifs shown have been omitted.

**Figure 9:**
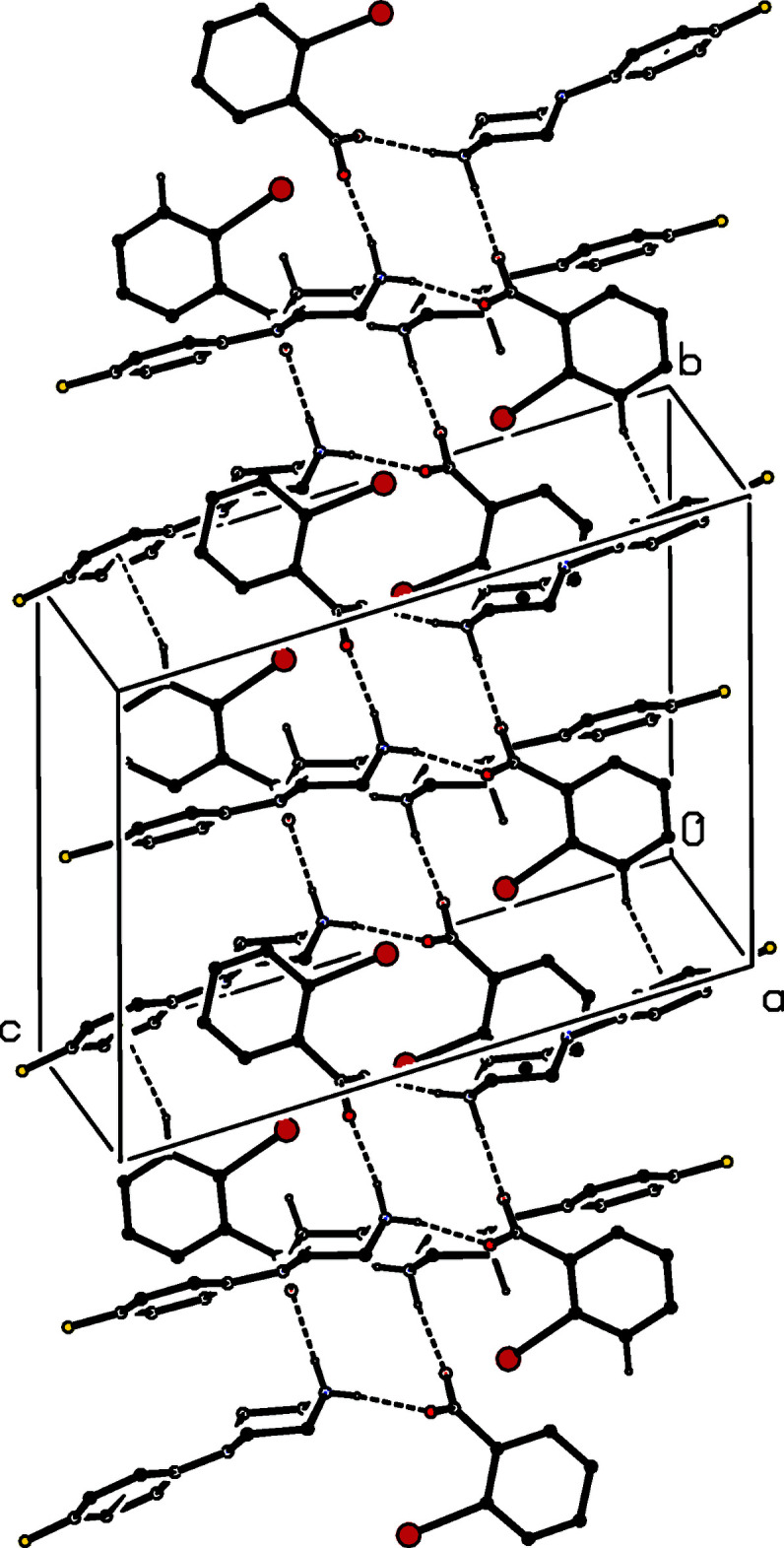
Part of the crystal structure of compound (III) showing the formation of a mol­ecular ribbon of edge-fused rings running parallel to the [010] direction. Hydrogen bonds are drawn as dashed lines and, for the sake of clarity, the H atoms bonded to those C atoms which are not involved in the motifs shown have been omitted.

**Figure 10:**
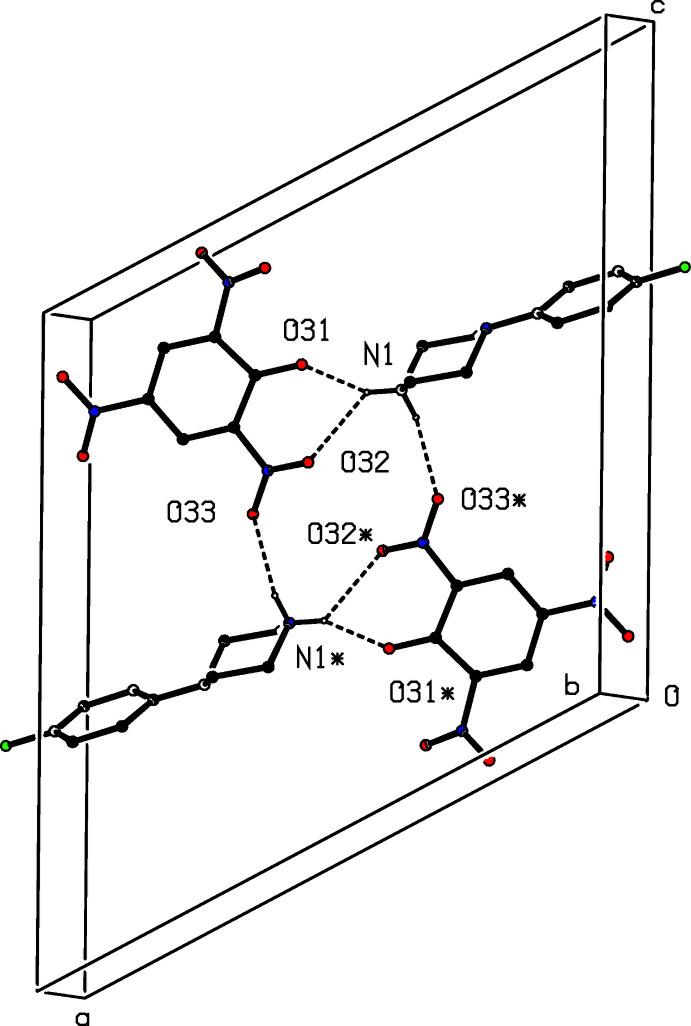
Part of the crystal structure of compound (IV) showing the formation of a centrosymmetric four-ion aggregate containing 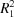
(6), 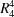
(12) and 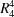
(16) ring types. Hydrogen bonds are drawn as dashed lines and, for the sake of clarity, the H atoms bonded to C atoms have been omitted. The atoms marked with an asterisk (*) are at the symmetry position (1 − *x*, 1 − *y*, 1 − *z*).

The component ions in compound (IV) are linked by a three-centre (bifurcated) N—H⋯(O)_2_ hydrogen bond within the selected asymmetric unit (Fig. 4 ▸, Table 1 ▸), while a two centre N—H⋯O hydrogen bond links the ions to form a centrosymmetric four-ion aggregate, in which rings of 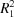
(6), 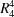
(12) and 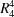
(16) types can be identified (Fig. 10 ▸): it is inter­esting to note the occurrence of the 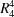
(12) ring type, exactly as in the structures of compounds (II) and (III). The structure of compound (IV) contains several short C—H⋯O contacts, but in all of these contacts the C—H⋯O angle is close to 140° (Table 1 ▸), so that their structural significance is likely to be minimal, at best (Wood *et al.*, 2009 ▸).

**Figure 11:**
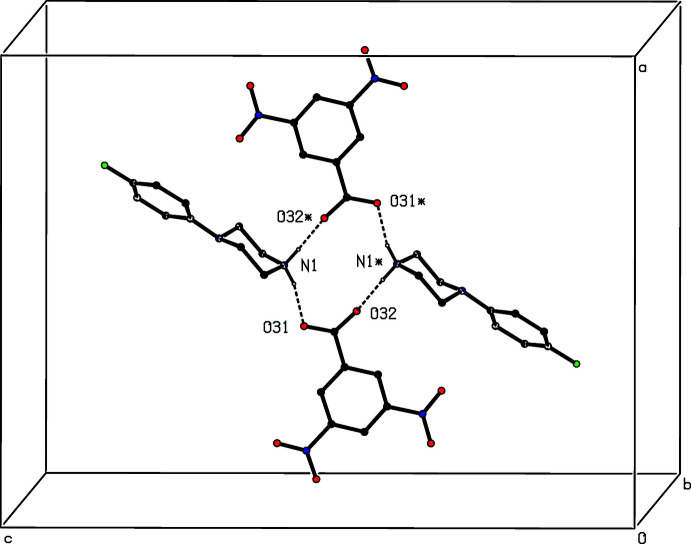
Part of the crystal structure of compound (V) showing the formation of a centrosymmetric four-ion 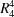
(12) aggregate. Hydrogen bonds are drawn as dashed lines and, for the sake of clarity, the H atoms bonded to C atoms have been omitted. The atoms marked with an asterisk (*) are at the symmetry position (1 − *x*, 1 − *y*, 1 − *z*).

**Figure 12:**
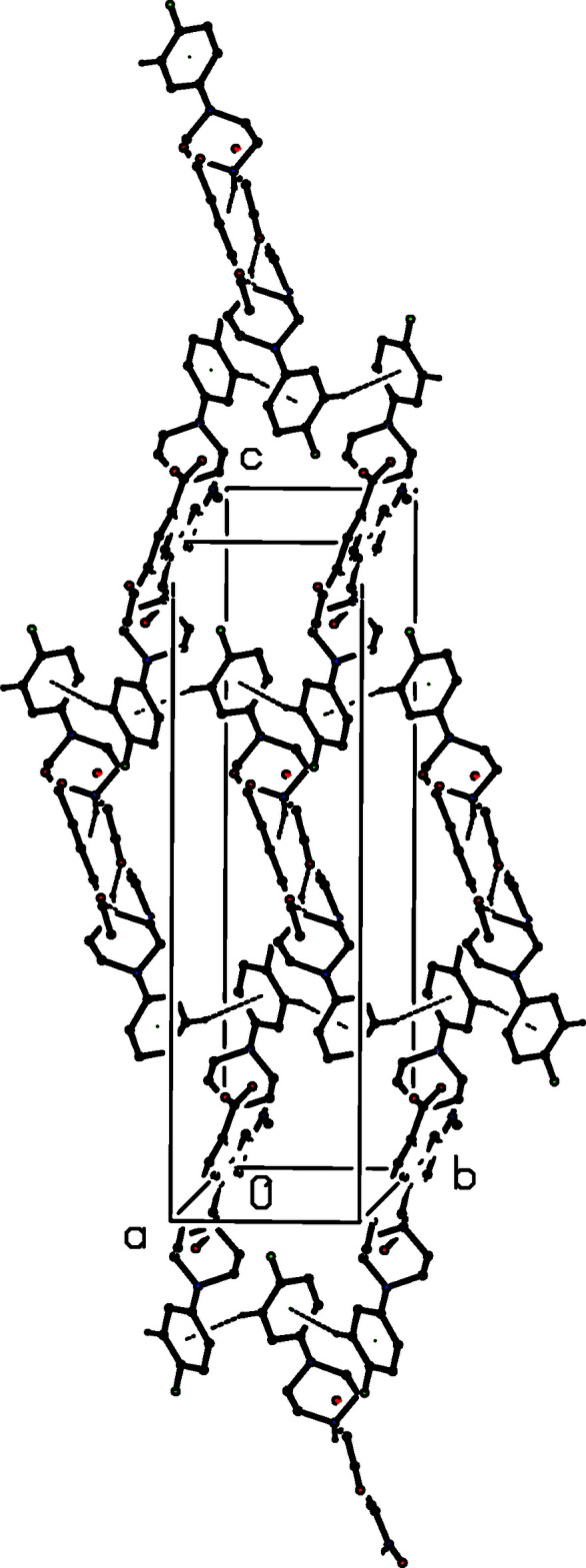
Part of the crystal structure of compound (V) showing the formation of a sheet lying parallel to (10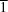) and formed from N—H⋯O and C—H⋯π(arene) hydrogen bonds, drawn as dashed lines. For the sake of clarity, the H atoms not involved in the motifs shown have been omitted.

**Figure 13:**
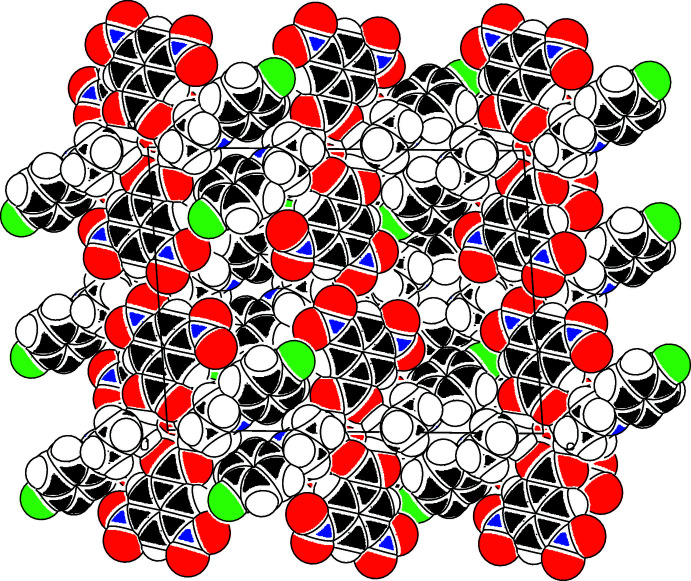
A space-filling projection down [010] of part of the crystal structure of compound (V) showing the formation of narrow channels parallel to the twofold rotation axes.

Inversion-related ion pairs in compound (V) form the same type of 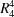
(12) motif (Fig. 11 ▸) as previously seen in each of compounds (II)–(IV). In addition, an almost linear C—H⋯π(arene) hydrogen bond links these four-ion aggregates into a sheet lying parallel to (10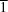) (Fig. 12 ▸). A second sheet of this type, related to the first by the twofold rotation axes, also passes through each unit cell, but there are no direction-specific inter­actions between adjacent sheets. However, the stacking of the sheets leaves void space in the form of narrow channels lying along the twofold axes (Fig. 13 ▸). As noted above (§ 2), the channels appear to contain solvent mol­ecules, which are disordered and/or mobile.

Thus the cyclic 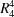
(12) motif can be identified in some form in each of compounds (II)–(V), although such rings are centrosymmetric in each of (II), (IV) and (V), but non-centrosymmetric in (III), and they are explicit in (II), (III) and (V) (Figs. 3 ▸, 7 ▸, 11 ▸), but masked within a more complex four-ion aggregate in (IV) (Fig. 10 ▸).

## Database survey   

It is of inter­est briefly to compare the structures reported here with those of some closely related compounds. One obvious comparison is between the monohydrated compound (I) reported here and a series of isostructural salts (benzoate, 4-fluoro­benzoate, 4-chloro­benzoate and 4-bromo­benzoate) of MeOPP [compounds (VI)–(IX)], all of which crystallize as monohydrates (Kiran Kumar *et al.*, 2019 ▸). Compounds (VI)–(IX) all form a chain of centrosymmetric 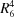
(12) and 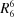
(16) rings comparable to that found here in compound (I). It is important to emphasize, however, that (I) is not isostructural with (VI)–(IX); thus, although the repeat vectors for the unit cell of (I) are somewhat similar to those in (VI)–(IX), the inter-axial angles in (I) are all greater than 90°, whereas in (VI)–(IX) they are consistently less than 90°.

The picrate salt of MeOPP (X) (Kiran Kumar *et al.*, 2020 ▸) is analogous to the picrate salt (IV) formed by 4-FPP, and both show the same pattern of electronic delocalization within the anion. While (IV) and (X) both crystallize in space group *P*2_1_/*n*, they are by no means isomorphous, and in (X) two of the nitro groups exhibit disorder. Whereas the N—H⋯O hydrogen bonds in (IV) generate a dimeric structure (Fig. 10 ▸), in (X) they generate a chain of rings, and adjacent chains are linked by a C—H⋯π(arene) hydrogen bond to form a sheet parallel to (001) (Kiran Kumar *et al.*, 2020 ▸). Similar delocalization is apparent in the anion of the 5-hy­droxy-3,5-di­nitro­benzoate salts of both 4-FPP (XI) (Harish Chinthal *et al.*, 2020 ▸) and MeOPP (XII) (Kiran Kumar *et al.*, 2019 ▸). However, in (XI) the ions are linked into a chain of 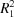
(4) and 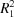
(6) rings by two independent three-centre N—H⋯(O)_2_ hydrogen bonds, while in (XII) a combination of N—H⋯O and C—H⋯O hydrogen bonds generates a chain of alternating 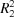
(10) and 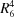
(16) rings, with the chains further linked into a three-dimensional framework structure by a single C—H⋯π(arene) hydrogen bond.

## Synthesis and crystallization   

All starting materials were obtained commercially, and all were used as received: solutions of *N*-(4-fluoro­phen­yl)piperazine (100 mg, 0.55 mol) in methanol (10 ml) were mixed with solutions of the appropriate acids (0.55 mol) in methanol (10 ml), *viz*. 2-fluoro­benzoic acid (77.1 mg) for (I), 2-bromo­benzoic acid (110.6 mg) for (II), 2-iodo­benzoic acid (136.4 mg) for (III), picric acid (126 mg) for (IV) and 3,5-di­nitro­benzoic acid (116.7 mg) for (V). The corresponding pairs of solution were mixed and then briefly held at 323 K, before being set aside to crystallize. After two days at room temperature, the resulting solid products were collected by filtration, dried in air and then crystallized, at ambient temperature and in the presence of air from mixture of ethyl acetate and acetone (initial composition 9:1, *v*/*v*) for (I), or ethyl acetate and methanol [initial composition 9:1, *v*/*v* for (II)–(IV), and 1:1 *v*/*v* for (V)]. M.p. (I) 349–351 K, (II) 411–414 K, (III) 409–411 K, (IV) 405–410 K, and (V) 426–430 K. Despite repeated efforts, we have been unable to obtain satisfactory crystals of the 2-chloro­benzoate salt, using solvents such as aceto­nitrile, acetone, ethyl acetate or methanol, and a number of mixtures of such solvents.

## Refinement   

Crystal data, data collection and refinement details are summarized in Table 2 ▸. All H atoms, apart from those of the partial-occupancy water mol­ecule in compound (II), were located in difference maps. The H atoms bonded to C atoms were then treated as riding atoms in geometrically idealized positions with C—H distances of 0.93 Å (aromatic) or 0.97 Å (CH_2_), and with *U*_iso_(H) = 1.2*U*_eq_(C). For the H atoms bonded to N atoms, the atomic coordinates were refined with *U*_iso_(H) = 1.2*U*_eq_(N) giving the N—H distances shown in Table 1 ▸. Before the final refinements for compound (V), one low-angle reflection, (110), which had been attenuated by the beam stop, and one bad outlier reflection, (004), were removed from the data set. It was not possible to reliably locate in difference maps the H atoms of the partial-occupancy water mol­ecule in (II); hence they were included in calculated positions, riding at 0.90 Å from the atom O41, at positions calculated by inter­polation along the relevant O⋯O vectors, with *U*_iso_(H) = 1.5*U*_eq_(O): the water atom O41 was refined isotropically and the refined occupancy of the water mol­ecule was 0.353 (8). The largest maximum and minimum in the final difference map for (III), +2.92 and −2.00 e Å^-3^, respectively, were both close to atom I232, at distances of 0.92 and 0.77 Å, respectively. Examination of the difference map for compound (V) after conventional refinement showed the presence of two significant peaks, one of 2.98 e Å^−3^ lying on a twofold rotation axis, at (0.5, −0.0533, 0.75), and the other, 1.94 e Å^−3^, lying in a general position at (0.534, 0.882, 0.760). No plausible solvent model could be developed based upon these two maxima and, accordingly, the data at this stage were subjected to the SQUEEZE procedure (Spek, 2015 ▸) prior to the final refinements, and it is the results of that refinement which are reported here for compound (V).

## Supplementary Material

Crystal structure: contains datablock(s) global, I, II, III, IV, V. DOI: 10.1107/S2056989020008749/hb7928sup1.cif

Structure factors: contains datablock(s) I. DOI: 10.1107/S2056989020008749/hb7928Isup2.hkl

Structure factors: contains datablock(s) II. DOI: 10.1107/S2056989020008749/hb7928IIsup3.hkl

Structure factors: contains datablock(s) III. DOI: 10.1107/S2056989020008749/hb7928IIIsup4.hkl

Structure factors: contains datablock(s) IV. DOI: 10.1107/S2056989020008749/hb7928IVsup5.hkl

Structure factors: contains datablock(s) V. DOI: 10.1107/S2056989020008749/hb7928Vsup6.hkl

Supporting information file. DOI: 10.1107/S2056989020008749/hb7928Isup7.cml

Supporting information file. DOI: 10.1107/S2056989020008749/hb7928IIIsup8.cml

Supporting information file. DOI: 10.1107/S2056989020008749/hb7928IVsup9.cml

Supporting information file. DOI: 10.1107/S2056989020008749/hb7928Vsup10.cml

CCDC references: 2012924, 2012923, 2012922, 2012921, 2012920

Additional supporting information:  crystallographic information; 3D view; checkCIF report

## Figures and Tables

**Figure 5:**
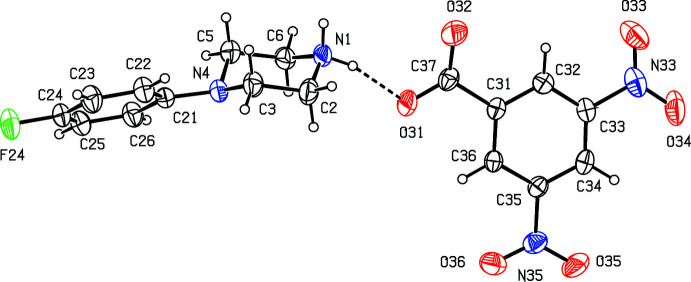
The independent components of compound (V) showing the atom-labeling scheme and the hydrogen bonds, drawn as dashed lines, within the selected asymmetric unit. Displacement ellipsoids are drawn at the 30% probability level.

**Table 1:** Hydrogen bonds and short inter­molecular contacts (Å, °) for compounds (I)-(V) *Cg*1, *Cg*2 and *Cg*3 represent the centroids of the rings (C31–C36), (C221–C226) and (C21–C26), respectively.

*D*—H⋯*A*	*D*—H	H⋯*A*	*D*⋯*A*	*D*—H⋯*A*
I				
N1—H11⋯O31	0.975 (19)	1.773 (19)	2.7426 (18)	173.2 (14)
N1—H12⋯O41	0.970 (16)	1.793 (16)	2.749 (2)	167.9 (18)
O41—H41⋯O32^i^	0.84 (3)	1.95 (3)	2.7744 (19)	168 (2)
O41—H42⋯O31^ii^	0.89 (3)	1.80 (3)	2.6693 (18)	164 (2)
C3—H3*A*⋯O32^iii^	0.97	2.44	3.317 (3)	149
				
(II)				
N1—H11⋯O32	0.78 (4)	1.93 (4)	2.677 (4)	160 (3)
N1—H12⋯O3^i^	0.91 (3)	1.80 (3)	2.707 (4)	175 (3)
O41—H41⋯O31	0.90	1.76	2.661 (12)	179
O41—H42⋯O31^iii^	0.89	1.90	2.792 (12)	179
C35—H35⋯O41^iv^	0.93	2.38	3.257 (11)	157
C2—H2*A*⋯*Cg*1^v^	0.97	2.78	3.598 (3)	142
				
(III)				
N11—H111⋯O132	0.80 (6)	1.89 (6)	2.680 (7)	168 (6)
N11—H112⋯O231	0.90 (6)	1.87 (6)	2.758 (6)	171 (4)
N21—H211⋯O232	0.79 (6)	1.88 (6)	2.665 (6)	173 (6)
N21—H212⋯O131	0.85 (7)	1.87 (7)	2.714 (6)	179 (8)
C13—H13*A*⋯O131^i^	0.97	2.50	3.396 (7)	154
C133—H133⋯*Cg*2^vi^	0.93	2.69	3.433 (6)	137
				
(IV)				
N1—H11⋯O31	0.91 (3)	1.85 (2)	2.687 (2)	153 (2)
N1—H11⋯O32	0.91 (3)	2.46 (3)	3.103 (3)	128.2 (17)
N1—H12⋯O33^i^	0.84 (2)	2.35 (2)	3.035 (2)	138.5 (18)
C5—H5*A*⋯O36^vii^	0.97	2.49	3.318 (3)	144
C6—H6*B*⋯O34^viii^	0.97	2.40	3.207 (3)	140
C25—H25⋯O37^ix^	0.93	2.58	3.365 (3)	142
				
(V)				
N1—H11⋯O31	0.94 (3)	1.77 (3)	2.681 (3)	164 (3)
N1—H11⋯O32^i^	0.95 (3)	1.80 (3)	2.731 (3)	165 (3)
C23—H23⋯*Cg*3^viii^	0.93	2.88	3.787 (3)	167

Symmetry codes: (i) 1 − *x*, 1 − *y*, 1 − *z*; (ii) −*x*, 1 − *y*, 1 − *z*; (iii) 1 − *x*, −*y*, 1 − *z*; (iv) 1 + *x*, *y*, *z*; (v) −1 + *x*, *y*, *z*; (vi) *x*, 1 + *y*, *z*; (vii) −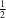 + *x*, 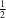 − *y*, −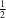 + *z*; (viii) 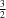 − *x*, −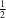 + *y*, 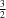 − *z*; (ix) 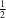 − *x*, −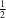 + *y*, 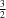 − *z*.

**Table 2:** Experimental details

	(I)	(II)	(III)	(IV)	(V)
Crystal data					
Chemical formula	C_10_H_14_FN_2_^+^·C_7_H_4_FO_2_^−^·H_2_O	C_10_H_14_FN_2_^+^·C_7_H_4_BrO_2_^−^·0.353H_2_O	C_10_H_14_FN_2_^+^·C_7_H_4_IO_2_^−^	C_10_H_14_FN_2_^+^·C_6_H_2_N_3_O_7_^−^	C_10_H_14_FN_2_^+^·C_7_H_3_N_2_O_6_^−^
*M*_r_	338.35	387.59	428.23	409.34	392.34
Crystal system, space group	Triclinic, *P* 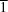	Triclinic, *P* 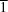	Triclinic, *P* 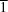	Monoclinic, *P*2_1_/*n*	Monoclinic, *C*2/*c*
Temperature (K)	293	293	293	293	293
*a*, *b*, *c* (Å)	6.5873 (5), 7.6616 (6), 17.3399 (9)	7.2584 (8), 9.347 (1), 13.767 (2)	9.8892 (3), 11.6831 (7), 16.4547 (9)	16.658 (2), 6.6734 (6), 17.553 (3)	19.871 (1), 7.3420 (7), 26.306 (2)
α, β, γ (°)	97.842 (6), 90.378 (6), 95.540 (6)	101.14 (1), 95.98 (1), 109.44 (1)	106.776 (5), 93.327 (4), 104.874 (4)	90, 117.84 (2), 90	90, 94.540 (8), 90
*V* (Å^3^)	862.72 (11)	849.72 (19)	1741.19 (16)	1725.4 (5)	3825.8 (5)
*Z*	2	2	4	4	8
Radiation type	Mo *K*α	Mo *K*α	Mo *K*α	Mo *K*α	Mo *K*α
μ (mm^−1^)	0.10	2.44	1.86	0.13	0.11
Crystal size (mm)	0.46 × 0.40 × 0.14	0.50 × 0.48 × 0.44	0.50 × 0.50 × 0.48	0.48 × 0.40 × 0.40	0.46 × 0.42 × 0.22

Data collection					
Diffractometer	Oxford Diffraction Xcalibur with Sapphire CCD	Oxford Diffraction Xcalibur with Sapphire CCD	Oxford Diffraction Xcalibur with Sapphire CCD	Oxford Diffraction Xcalibur with Sapphire CCD	Oxford Diffraction Xcalibur with Sapphire CCD
Absorption correction	Multi-scan (*CrysAlis RED*; Oxford Diffraction, 2009 ▸)	Multi-scan (*CrysAlis RED*; Oxford Diffraction, 2009 ▸)	Multi-scan (*CrysAlis RED*; Oxford Diffraction, 2009 ▸)	Multi-scan (*CrysAlis RED*; Oxford Diffraction, 2009 ▸)	Multi-scan (*CrysAlis RED*; Oxford Diffraction, 2009 ▸)
*T*_min_, *T*_max_	0.871, 0.986	0.257, 0.341	0.383, 0.411	0.816, 0.949	0.832, 0.976
No. of measured, independent and observed [*I* > 2σ(*I*)] reflections	5683, 3567, 2350	5475, 3582, 2775	12798, 7396, 5890	12153, 3865, 2922	7938, 4080, 2490
*R*_int_	0.012	0.026	0.015	0.019	0.017
(sin θ/λ)_max_ (Å^−1^)	0.629	0.650	0.651	0.656	0.654

Refinement					
*R*[*F* ^2^ > 2σ(*F* ^2^)], *wR*(*F* ^2^), *S*	0.045, 0.123, 1.01	0.043, 0.124, 1.10	0.047, 0.127, 1.06	0.045, 0.136, 1.05	0.056, 0.144, 1.07
No. of reflections	3567	3582	7396	3865	4080
No. of parameters	229	219	427	269	259
H-atom treatment	H atoms treated by a mixture of independent and constrained refinement	H atoms treated by a mixture of independent and constrained refinement	H atoms treated by a mixture of independent and constrained refinement	H atoms treated by a mixture of independent and constrained refinement	H atoms treated by a mixture of independent and constrained refinement
Δρ_max_, Δρ_min_ (e Å^−3^)	0.20, −0.18	0.47, −0.77	2.92, −2.00	0.45, −0.27	0.19, −0.18

Computer programs: *CrysAlis CCD* (Oxford Diffraction, 2009 ▸), *CrysAlis RED* (Oxford Diffraction, 2009 ▸), *CrysAlis RED* (Oxford Diffraction, 2009 ▸, *SHELXT* (Sheldrick, 2015*a* ▸), *SHELXL2014* (Sheldrick, 2015*b* ▸) and *PLATON* (Spek, 2020 ▸).

## supplementary crystallographic information

### 4-(4-Fluorophenyl)piperazin-1-ium 2-fluorobenzoate monohydrate (I) Crystal data

**Table:**

C_10_H_14_FN_2_^+^·C_7_H_4_FO_2_^−^·H_2_O	*Z* = 2
*M**_r_* = 338.35	*F*(000) = 356
Triclinic, *P*1	*D*_x_ = 1.302 Mg m^−^^3^
*a* = 6.5873 (5) Å	Mo *K*α radiation, λ = 0.71073 Å
*b* = 7.6616 (6) Å	Cell parameters from 3684 reflections
*c* = 17.3399 (9) Å	θ = 3.1–27.8°
α = 97.842 (6)°	µ = 0.10 mm^−^^1^
β = 90.378 (6)°	*T* = 293 K
γ = 95.540 (6)°	Plate, yellow
*V* = 862.72 (11) Å^3^	0.46 × 0.40 × 0.14 mm

### 4-(4-Fluorophenyl)piperazin-1-ium 2-fluorobenzoate monohydrate (I) Data collection

**Table:**

Oxford Diffraction Xcalibur with Sapphire CCD diffractometer	3567 independent reflections
Radiation source: Enhance (Mo) X-ray Source	2350 reflections with *I* > 2σ(*I*)
Graphite monochromator	*R*_int_ = 0.012
ω scans	θ_max_ = 26.6°, θ_min_ = 3.1°
Absorption correction: multi-scan (CrysAlis RED; Oxford Diffraction, 2009)	*h* = −8→8
*T*_min_ = 0.871, *T*_max_ = 0.986	*k* = −9→9
5683 measured reflections	*l* = −21→21

### 4-(4-Fluorophenyl)piperazin-1-ium 2-fluorobenzoate monohydrate (I) Refinement

**Table:**

Refinement on *F*^2^	Primary atom site location: difference Fourier map
Least-squares matrix: full	Hydrogen site location: mixed
*R*[*F*^2^ > 2σ(*F*^2^)] = 0.045	H atoms treated by a mixture of independent and constrained refinement
*wR*(*F*^2^) = 0.123	*w* = 1/[σ^2^(*F*_o_^2^) + (0.0551*P*)^2^ + 0.1332*P*] where *P* = (*F*_o_^2^ + 2*F*_c_^2^)/3
*S* = 1.01	(Δ/σ)_max_ = 0.001
3567 reflections	Δρ_max_ = 0.20 e Å^−^^3^
229 parameters	Δρ_min_ = −0.18 e Å^−^^3^
0 restraints	

### 4-(4-Fluorophenyl)piperazin-1-ium 2-fluorobenzoate monohydrate (I) Special details

**Table:**

Geometry. All esds (except the esd in the dihedral angle between two l.s. planes) are estimated using the full covariance matrix. The cell esds are taken into account individually in the estimation of esds in distances, angles and torsion angles; correlations between esds in cell parameters are only used when they are defined by crystal symmetry. An approximate (isotropic) treatment of cell esds is used for estimating esds involving l.s. planes.

### 4-(4-Fluorophenyl)piperazin-1-ium 2-fluorobenzoate monohydrate (I) Fractional atomic coordinates and isotropic or equivalent isotropic displacement parameters (Å^2^)

**Table:**

	*x*	*y*	*z*	*U*_iso_*/*U*_eq_	
N1	0.1956 (2)	0.2775 (2)	0.55321 (8)	0.0526 (4)	
H11	0.128 (3)	0.248 (2)	0.5023 (11)	0.063*	
H12	0.261 (3)	0.397 (2)	0.5549 (10)	0.063*	
C2	0.3525 (3)	0.1578 (2)	0.56592 (10)	0.0613 (5)	
H2A	0.4556	0.1635	0.5264	0.074*	
H2B	0.2900	0.0369	0.5616	0.074*	
C3	0.4500 (3)	0.2110 (3)	0.64506 (10)	0.0600 (5)	
H3A	0.5502	0.1302	0.6533	0.072*	
H3B	0.5204	0.3290	0.6478	0.072*	
N4	0.2988 (2)	0.20918 (17)	0.70635 (8)	0.0505 (3)	
C5	0.1372 (3)	0.3200 (2)	0.69427 (10)	0.0564 (4)	
H5A	0.1933	0.4430	0.7007	0.068*	
H5B	0.0341	0.3071	0.7333	0.068*	
C6	0.0396 (3)	0.2714 (3)	0.61423 (10)	0.0601 (5)	
H6A	−0.0311	0.1533	0.6098	0.072*	
H6B	−0.0600	0.3533	0.6068	0.072*	
C21	0.3776 (3)	0.2198 (2)	0.78315 (9)	0.0529 (4)	
C22	0.5444 (3)	0.1322 (3)	0.79801 (12)	0.0717 (5)	
H22	0.6084	0.0703	0.7567	0.086*	
C23	0.6182 (4)	0.1350 (3)	0.87334 (14)	0.0871 (7)	
H23	0.7307	0.0756	0.8824	0.105*	
C24	0.5257 (4)	0.2242 (3)	0.93308 (12)	0.0856 (7)	
F24	0.5993 (3)	0.2265 (2)	1.00693 (8)	0.1322 (6)	
C25	0.3638 (4)	0.3123 (3)	0.92138 (12)	0.0915 (7)	
H25	0.3023	0.3737	0.9635	0.110*	
C26	0.2891 (3)	0.3115 (3)	0.84663 (11)	0.0757 (6)	
H26	0.1778	0.3734	0.8388	0.091*	
C31	0.1143 (2)	0.23893 (19)	0.27760 (9)	0.0460 (4)	
C32	0.2604 (3)	0.3128 (2)	0.23158 (11)	0.0608 (5)	
F32	0.45031 (17)	0.36529 (19)	0.26186 (8)	0.0980 (4)	
C33	0.2228 (3)	0.3407 (3)	0.15683 (13)	0.0800 (6)	
H33	0.3239	0.3951	0.1285	0.096*	
C34	0.0325 (4)	0.2866 (3)	0.12448 (12)	0.0806 (6)	
H34	0.0052	0.3004	0.0731	0.097*	
C35	−0.1176 (3)	0.2123 (3)	0.16775 (12)	0.0757 (6)	
H35	−0.2466	0.1763	0.1457	0.091*	
C36	−0.0777 (3)	0.1908 (2)	0.24373 (10)	0.0605 (5)	
H36	−0.1816	0.1431	0.2729	0.073*	
C37	0.1585 (2)	0.2067 (2)	0.35934 (10)	0.0485 (4)	
O31	0.01691 (18)	0.21983 (18)	0.40748 (7)	0.0682 (4)	
O32	0.32700 (18)	0.16218 (18)	0.37573 (8)	0.0718 (4)	
O41	0.3280 (2)	0.63088 (18)	0.56039 (9)	0.0707 (4)	
H41	0.430 (4)	0.685 (3)	0.5852 (15)	0.106*	
H42	0.224 (4)	0.688 (3)	0.5795 (14)	0.106*	

### 4-(4-Fluorophenyl)piperazin-1-ium 2-fluorobenzoate monohydrate (I) Atomic displacement parameters (Å^2^)

**Table:**

	*U*^11^	*U*^22^	*U*^33^	*U*^12^	*U*^13^	*U*^23^
N1	0.0615 (9)	0.0535 (8)	0.0436 (8)	0.0059 (7)	−0.0030 (7)	0.0095 (6)
C2	0.0789 (12)	0.0603 (11)	0.0489 (10)	0.0238 (9)	0.0110 (9)	0.0097 (8)
C3	0.0601 (10)	0.0749 (12)	0.0513 (10)	0.0275 (9)	0.0088 (8)	0.0163 (8)
N4	0.0562 (8)	0.0558 (8)	0.0426 (8)	0.0151 (6)	0.0054 (6)	0.0114 (6)
C5	0.0524 (10)	0.0683 (11)	0.0512 (10)	0.0156 (8)	0.0070 (8)	0.0113 (8)
C6	0.0523 (10)	0.0705 (11)	0.0596 (11)	0.0083 (8)	0.0017 (8)	0.0157 (9)
C21	0.0637 (10)	0.0501 (9)	0.0461 (10)	0.0065 (8)	0.0014 (8)	0.0107 (7)
C22	0.0830 (13)	0.0771 (13)	0.0580 (12)	0.0253 (11)	−0.0088 (10)	0.0080 (9)
C23	0.1000 (17)	0.0901 (15)	0.0751 (15)	0.0248 (13)	−0.0274 (13)	0.0156 (12)
C24	0.1192 (19)	0.0878 (15)	0.0498 (12)	0.0060 (14)	−0.0209 (12)	0.0130 (11)
F24	0.1853 (16)	0.1527 (14)	0.0599 (8)	0.0244 (12)	−0.0440 (9)	0.0162 (8)
C25	0.120 (2)	0.1097 (18)	0.0467 (12)	0.0245 (16)	0.0060 (12)	0.0066 (11)
C26	0.0880 (14)	0.0932 (15)	0.0497 (12)	0.0259 (12)	0.0062 (10)	0.0108 (10)
C31	0.0482 (9)	0.0404 (8)	0.0491 (9)	0.0074 (7)	−0.0027 (7)	0.0029 (7)
C32	0.0513 (10)	0.0673 (11)	0.0624 (12)	−0.0003 (8)	−0.0023 (9)	0.0086 (9)
F32	0.0601 (7)	0.1385 (11)	0.0924 (9)	−0.0228 (7)	−0.0031 (6)	0.0283 (8)
C33	0.0815 (15)	0.0946 (16)	0.0662 (13)	−0.0028 (12)	0.0108 (11)	0.0267 (11)
C34	0.0925 (16)	0.0986 (16)	0.0546 (12)	0.0100 (13)	−0.0069 (11)	0.0236 (11)
C35	0.0707 (13)	0.0935 (15)	0.0627 (13)	−0.0016 (11)	−0.0201 (10)	0.0178 (11)
C36	0.0571 (10)	0.0673 (11)	0.0570 (11)	−0.0021 (8)	−0.0084 (8)	0.0146 (8)
C37	0.0472 (9)	0.0447 (9)	0.0521 (10)	0.0063 (7)	−0.0065 (8)	0.0009 (7)
O31	0.0538 (7)	0.1020 (10)	0.0504 (7)	0.0164 (7)	−0.0032 (6)	0.0098 (6)
O32	0.0586 (8)	0.0929 (10)	0.0695 (9)	0.0283 (7)	−0.0068 (6)	0.0163 (7)
O41	0.0574 (8)	0.0642 (8)	0.0923 (11)	0.0069 (6)	0.0025 (7)	0.0166 (7)

### 4-(4-Fluorophenyl)piperazin-1-ium 2-fluorobenzoate monohydrate (I) Geometric parameters (Å, º)

**Table:**

N1—C2	1.481 (2)	C23—H23	0.9300
N1—C6	1.482 (2)	C24—C25	1.345 (3)
N1—H11	0.974 (19)	C24—F24	1.364 (2)
N1—H12	0.973 (18)	C25—C26	1.382 (3)
C2—C3	1.499 (2)	C25—H25	0.9300
C2—H2A	0.9700	C26—H26	0.9300
C2—H2B	0.9700	C31—C32	1.383 (2)
C3—N4	1.462 (2)	C31—C36	1.386 (2)
C3—H3A	0.9700	C31—C37	1.503 (2)
C3—H3B	0.9700	C32—F32	1.356 (2)
N4—C21	1.415 (2)	C32—C33	1.367 (3)
N4—C5	1.455 (2)	C33—C34	1.373 (3)
C5—C6	1.510 (2)	C33—H33	0.9300
C5—H5A	0.9700	C34—C35	1.372 (3)
C5—H5B	0.9700	C34—H34	0.9300
C6—H6A	0.9700	C35—C36	1.377 (3)
C6—H6B	0.9700	C35—H35	0.9300
C21—C22	1.382 (2)	C36—H36	0.9300
C21—C26	1.386 (2)	C37—O32	1.2347 (18)
C22—C23	1.387 (3)	C37—O31	1.2580 (19)
C22—H22	0.9300	O41—H41	0.84 (3)
C23—C24	1.344 (3)	O41—H42	0.89 (3)
			
C2—N1—C6	109.84 (13)	C21—C22—H22	119.4
C2—N1—H11	112.8 (10)	C23—C22—H22	119.4
C6—N1—H11	108.9 (10)	C24—C23—C22	119.5 (2)
C2—N1—H12	109.1 (10)	C24—C23—H23	120.3
C6—N1—H12	110.1 (10)	C22—C23—H23	120.3
H11—N1—H12	106.0 (15)	C23—C24—C25	121.4 (2)
N1—C2—C3	109.97 (14)	C23—C24—F24	119.1 (2)
N1—C2—H2A	109.7	C25—C24—F24	119.5 (2)
C3—C2—H2A	109.7	C24—C25—C26	119.8 (2)
N1—C2—H2B	109.7	C24—C25—H25	120.1
C3—C2—H2B	109.7	C26—C25—H25	120.1
H2A—C2—H2B	108.2	C25—C26—C21	121.0 (2)
N4—C3—C2	111.44 (15)	C25—C26—H26	119.5
N4—C3—H3A	109.3	C21—C26—H26	119.5
C2—C3—H3A	109.3	C32—C31—C36	116.39 (16)
N4—C3—H3B	109.3	C32—C31—C37	122.84 (15)
C2—C3—H3B	109.3	C36—C31—C37	120.76 (15)
H3A—C3—H3B	108.0	F32—C32—C33	117.41 (17)
C21—N4—C5	117.11 (13)	F32—C32—C31	119.16 (16)
C21—N4—C3	115.84 (14)	C33—C32—C31	123.41 (17)
C5—N4—C3	111.76 (12)	C32—C33—C34	118.53 (19)
N4—C5—C6	111.74 (14)	C32—C33—H33	120.7
N4—C5—H5A	109.3	C34—C33—H33	120.7
C6—C5—H5A	109.3	C35—C34—C33	120.21 (19)
N4—C5—H5B	109.3	C35—C34—H34	119.9
C6—C5—H5B	109.3	C33—C34—H34	119.9
H5A—C5—H5B	107.9	C34—C35—C36	120.10 (19)
N1—C6—C5	110.66 (14)	C34—C35—H35	120.0
N1—C6—H6A	109.5	C36—C35—H35	120.0
C5—C6—H6A	109.5	C35—C36—C31	121.31 (18)
N1—C6—H6B	109.5	C35—C36—H36	119.3
C5—C6—H6B	109.5	C31—C36—H36	119.3
H6A—C6—H6B	108.1	O32—C37—O31	122.88 (16)
C22—C21—C26	117.06 (17)	O32—C37—C31	119.41 (15)
C22—C21—N4	120.50 (15)	O31—C37—C31	117.65 (14)
C26—C21—N4	122.41 (16)	H41—O41—H42	104 (2)
C21—C22—C23	121.2 (2)		
			
C6—N1—C2—C3	−58.4 (2)	C24—C25—C26—C21	0.5 (4)
N1—C2—C3—N4	57.70 (19)	C22—C21—C26—C25	−0.9 (3)
C2—C3—N4—C21	166.90 (14)	N4—C21—C26—C25	176.88 (19)
C2—C3—N4—C5	−55.50 (19)	C36—C31—C32—F32	178.92 (16)
C21—N4—C5—C6	−169.07 (14)	C37—C31—C32—F32	−2.6 (2)
C3—N4—C5—C6	53.91 (19)	C36—C31—C32—C33	0.7 (3)
C2—N1—C6—C5	57.07 (19)	C37—C31—C32—C33	179.14 (17)
N4—C5—C6—N1	−54.95 (19)	F32—C32—C33—C34	179.15 (19)
C5—N4—C21—C22	−173.58 (16)	C31—C32—C33—C34	−2.6 (3)
C3—N4—C21—C22	−38.3 (2)	C32—C33—C34—C35	2.4 (3)
C5—N4—C21—C26	8.7 (2)	C33—C34—C35—C36	−0.3 (3)
C3—N4—C21—C26	143.97 (18)	C34—C35—C36—C31	−1.7 (3)
C26—C21—C22—C23	0.6 (3)	C32—C31—C36—C35	1.5 (3)
N4—C21—C22—C23	−177.22 (18)	C37—C31—C36—C35	−177.02 (16)
C21—C22—C23—C24	0.1 (4)	C32—C31—C37—O32	−35.4 (2)
C22—C23—C24—C25	−0.6 (4)	C36—C31—C37—O32	143.03 (17)
C22—C23—C24—F24	−179.9 (2)	C32—C31—C37—O31	147.48 (16)
C23—C24—C25—C26	0.3 (4)	C36—C31—C37—O31	−34.1 (2)
F24—C24—C25—C26	179.6 (2)		

### 4-(4-Fluorophenyl)piperazin-1-ium 2-fluorobenzoate monohydrate (I) Hydrogen-bond geometry (Å, º)

**Table:**

*D*—H···*A*	*D*—H	H···*A*	*D*···*A*	*D*—H···*A*
N1—H11···O31	0.975 (19)	1.773 (19)	2.7426 (18)	173.2 (14)
N1—H12···O41	0.970 (16)	1.793 (16)	2.749 (2)	167.9 (18)
O41—H41···O32^i^	0.84 (3)	1.95 (3)	2.7744 (19)	168 (2)
O41—H42···O31^ii^	0.89 (3)	1.80 (3)	2.6693 (18)	164 (2)
C3—H3*A*···O32^iii^	0.97	2.44	3.317 (3)	149

Symmetry codes: (i) −*x*+1, −*y*+1, −*z*+1; (ii) −*x*, −*y*+1, −*z*+1; (iii) −*x*+1, −*y*, −*z*+1.

### 4-(4-Fluorophenyl)piperazin-1-ium 2-bromobenzoate 0.353-hydrate (II) Crystal data

**Table:**

C_10_H_14_FN_2_^+^·C_7_H_4_BrO_2_^−^·0.353H_2_O	*Z* = 2
*M**_r_* = 387.59	*F*(000) = 395.1
Triclinic, *P*1	*D*_x_ = 1.515 Mg m^−^^3^
*a* = 7.2584 (8) Å	Mo *K*α radiation, λ = 0.71073 Å
*b* = 9.347 (1) Å	Cell parameters from 3585 reflections
*c* = 13.767 (2) Å	θ = 3.0–27.6°
α = 101.14 (1)°	µ = 2.44 mm^−^^1^
β = 95.98 (1)°	*T* = 293 K
γ = 109.44 (1)°	Block, orange
*V* = 849.72 (19) Å^3^	0.50 × 0.48 × 0.44 mm

### 4-(4-Fluorophenyl)piperazin-1-ium 2-bromobenzoate 0.353-hydrate (II) Data collection

**Table:**

Oxford Diffraction Xcalibur with Sapphire CCD diffractometer	3582 independent reflections
Radiation source: Enhance (Mo) X-ray Source	2775 reflections with *I* > 2σ(*I*)
Graphite monochromator	*R*_int_ = 0.026
ω scans	θ_max_ = 27.5°, θ_min_ = 3.0°
Absorption correction: multi-scan (CrysAlis RED; Oxford Diffraction, 2009)	*h* = −4→9
*T*_min_ = 0.257, *T*_max_ = 0.341	*k* = −12→11
5475 measured reflections	*l* = −16→17

### 4-(4-Fluorophenyl)piperazin-1-ium 2-bromobenzoate 0.353-hydrate (II) Refinement

**Table:**

Refinement on *F*^2^	Primary atom site location: difference Fourier map
Least-squares matrix: full	Hydrogen site location: mixed
*R*[*F*^2^ > 2σ(*F*^2^)] = 0.043	H atoms treated by a mixture of independent and constrained refinement
*wR*(*F*^2^) = 0.124	*w* = 1/[σ^2^(*F*_o_^2^) + (0.0776*P*)^2^] where *P* = (*F*_o_^2^ + 2*F*_c_^2^)/3
*S* = 1.10	(Δ/σ)_max_ < 0.001
3582 reflections	Δρ_max_ = 0.47 e Å^−^^3^
219 parameters	Δρ_min_ = −0.77 e Å^−^^3^
0 restraints	

### 4-(4-Fluorophenyl)piperazin-1-ium 2-bromobenzoate 0.353-hydrate (II) Special details

**Table:**

Geometry. All esds (except the esd in the dihedral angle between two l.s. planes) are estimated using the full covariance matrix. The cell esds are taken into account individually in the estimation of esds in distances, angles and torsion angles; correlations between esds in cell parameters are only used when they are defined by crystal symmetry. An approximate (isotropic) treatment of cell esds is used for estimating esds involving l.s. planes.

### 4-(4-Fluorophenyl)piperazin-1-ium 2-bromobenzoate 0.353-hydrate (II) Fractional atomic coordinates and isotropic or equivalent isotropic displacement parameters (Å^2^)

**Table:**

	*x*	*y*	*z*	*U*_iso_*/*U*_eq_	Occ. (<1)
N1	0.4921 (4)	0.5765 (3)	0.36697 (18)	0.0441 (5)	
H11	0.577 (5)	0.549 (4)	0.386 (2)	0.053*	
H12	0.448 (5)	0.616 (4)	0.422 (2)	0.053*	
C2	0.3355 (5)	0.4415 (3)	0.2958 (2)	0.0553 (7)	
H2A	0.2812	0.3601	0.3304	0.066*	
H2B	0.3928	0.3996	0.2417	0.066*	
C3	0.1719 (4)	0.4880 (3)	0.2525 (2)	0.0516 (7)	
H3A	0.0718	0.3979	0.2054	0.062*	
H3B	0.1095	0.5246	0.3061	0.062*	
N4	0.2506 (3)	0.6110 (2)	0.20113 (16)	0.0398 (5)	
C5	0.4007 (4)	0.7471 (3)	0.2736 (2)	0.0512 (7)	
H5A	0.3388	0.7855	0.3267	0.061*	
H5B	0.4530	0.8300	0.2398	0.061*	
C6	0.5673 (4)	0.7068 (4)	0.3186 (2)	0.0520 (7)	
H6A	0.6373	0.6777	0.2665	0.062*	
H6B	0.6605	0.7976	0.3682	0.062*	
C21	0.1090 (4)	0.6461 (3)	0.1414 (2)	0.0402 (6)	
C22	−0.0941 (4)	0.5824 (3)	0.1394 (2)	0.0516 (7)	
H22	−0.1410	0.5151	0.1804	0.062*	
C23	−0.2270 (5)	0.6168 (4)	0.0782 (3)	0.0658 (9)	
H23	−0.3628	0.5720	0.0767	0.079*	
C24	−0.1579 (6)	0.7161 (5)	0.0204 (2)	0.0689 (10)	
F24	−0.2891 (5)	0.7538 (4)	−0.0391 (2)	0.1093 (9)	
C25	0.0363 (7)	0.7830 (5)	0.0199 (3)	0.0724 (10)	
H25	0.0796	0.8530	−0.0200	0.087*	
C26	0.1726 (5)	0.7464 (4)	0.0798 (2)	0.0556 (7)	
H26	0.3074	0.7898	0.0784	0.067*	
C31	0.8354 (4)	0.2190 (3)	0.3694 (2)	0.0373 (5)	
C32	0.7930 (4)	0.1403 (3)	0.2693 (2)	0.0402 (6)	
Br32	0.59370 (5)	0.15933 (4)	0.17735 (2)	0.06077 (16)	
C33	0.8889 (5)	0.0410 (3)	0.2323 (3)	0.0552 (8)	
H33	0.8575	−0.0114	0.1646	0.066*	
C34	1.0298 (5)	0.0214 (4)	0.2964 (3)	0.0628 (9)	
H34	1.0970	−0.0431	0.2721	0.075*	
C35	1.0731 (5)	0.0959 (4)	0.3964 (3)	0.0622 (9)	
H35	1.1678	0.0808	0.4400	0.075*	
C36	0.9752 (4)	0.1941 (4)	0.4325 (2)	0.0529 (7)	
H36	1.0047	0.2439	0.5006	0.064*	
C37	0.7364 (4)	0.3310 (3)	0.41091 (19)	0.0417 (6)	
O31	0.6248 (4)	0.2899 (3)	0.4701 (2)	0.0826 (8)	
O32	0.7754 (3)	0.4539 (2)	0.38357 (18)	0.0584 (6)	
O41	0.4360 (13)	0.0019 (13)	0.4890 (8)	0.101 (4)*	0.353 (8)
H41	0.4989	0.0990	0.4825	0.152*	0.353 (8)
H42	0.4159	−0.0920	0.5015	0.152*	0.353 (8)

### 4-(4-Fluorophenyl)piperazin-1-ium 2-bromobenzoate 0.353-hydrate (II) Atomic displacement parameters (Å^2^)

**Table:**

	*U*^11^	*U*^22^	*U*^33^	*U*^12^	*U*^13^	*U*^23^
N1	0.0506 (14)	0.0534 (14)	0.0440 (12)	0.0320 (12)	0.0205 (11)	0.0181 (10)
C2	0.0616 (18)	0.0406 (15)	0.070 (2)	0.0195 (14)	0.0168 (15)	0.0247 (14)
C3	0.0439 (15)	0.0436 (16)	0.070 (2)	0.0094 (13)	0.0157 (14)	0.0283 (14)
N4	0.0430 (12)	0.0293 (10)	0.0464 (12)	0.0086 (9)	0.0126 (10)	0.0130 (9)
C5	0.0536 (17)	0.0360 (14)	0.0578 (17)	0.0061 (13)	0.0045 (14)	0.0180 (12)
C6	0.0431 (15)	0.0511 (17)	0.0627 (18)	0.0119 (13)	0.0139 (13)	0.0226 (14)
C21	0.0484 (15)	0.0343 (13)	0.0368 (12)	0.0163 (12)	0.0080 (11)	0.0037 (10)
C22	0.0509 (16)	0.0463 (16)	0.0535 (17)	0.0147 (14)	0.0064 (13)	0.0098 (13)
C23	0.0569 (19)	0.068 (2)	0.060 (2)	0.0223 (17)	−0.0068 (16)	−0.0029 (17)
C24	0.088 (3)	0.079 (2)	0.0451 (18)	0.051 (2)	−0.0058 (17)	0.0003 (17)
F24	0.134 (2)	0.143 (2)	0.0717 (15)	0.088 (2)	−0.0174 (15)	0.0237 (15)
C25	0.115 (3)	0.074 (2)	0.0461 (18)	0.050 (2)	0.0165 (19)	0.0253 (16)
C26	0.072 (2)	0.0548 (18)	0.0496 (16)	0.0277 (16)	0.0181 (14)	0.0207 (14)
C31	0.0367 (13)	0.0303 (12)	0.0491 (14)	0.0128 (10)	0.0180 (11)	0.0123 (10)
C32	0.0449 (14)	0.0278 (12)	0.0495 (14)	0.0115 (11)	0.0188 (11)	0.0104 (10)
Br32	0.0670 (2)	0.0549 (2)	0.0537 (2)	0.02015 (17)	0.00086 (15)	0.00658 (14)
C33	0.072 (2)	0.0381 (15)	0.0619 (18)	0.0243 (15)	0.0303 (16)	0.0081 (13)
C34	0.070 (2)	0.0430 (16)	0.095 (3)	0.0350 (16)	0.043 (2)	0.0215 (17)
C35	0.0556 (18)	0.0560 (19)	0.092 (3)	0.0325 (16)	0.0196 (17)	0.0318 (18)
C36	0.0561 (17)	0.0517 (17)	0.0558 (17)	0.0244 (14)	0.0125 (14)	0.0139 (13)
C37	0.0414 (14)	0.0411 (14)	0.0424 (14)	0.0172 (12)	0.0132 (11)	0.0025 (11)
O31	0.107 (2)	0.0798 (17)	0.0924 (19)	0.0535 (16)	0.0711 (17)	0.0299 (14)
O32	0.0564 (12)	0.0443 (11)	0.0858 (16)	0.0290 (10)	0.0240 (11)	0.0164 (11)

### 4-(4-Fluorophenyl)piperazin-1-ium 2-bromobenzoate 0.353-hydrate (II) Geometric parameters (Å, º)

**Table:**

N1—C6	1.473 (4)	C24—C25	1.340 (6)
N1—C2	1.476 (4)	C24—F24	1.369 (4)
N1—H11	0.78 (3)	C25—C26	1.393 (5)
N1—H12	0.92 (3)	C25—H25	0.9300
C2—C3	1.500 (4)	C26—H26	0.9300
C2—H2A	0.9700	C31—C36	1.376 (4)
C2—H2B	0.9700	C31—C32	1.379 (4)
C3—N4	1.453 (3)	C31—C37	1.514 (3)
C3—H3A	0.9700	C32—C33	1.388 (4)
C3—H3B	0.9700	C32—Br32	1.898 (3)
N4—C21	1.412 (3)	C33—C34	1.363 (5)
N4—C5	1.470 (4)	C33—H33	0.9300
C5—C6	1.492 (4)	C34—C35	1.368 (5)
C5—H5A	0.9700	C34—H34	0.9300
C5—H5B	0.9700	C35—C36	1.387 (4)
C6—H6A	0.9700	C35—H35	0.9300
C6—H6B	0.9700	C36—H36	0.9300
C21—C26	1.382 (4)	C37—O32	1.231 (3)
C21—C22	1.389 (4)	C37—O31	1.233 (3)
C22—C23	1.373 (4)	O41—O41^i^	0.962 (16)
C22—H22	0.9300	O41—H41	0.8970
C23—C24	1.344 (5)	O41—H42	0.8940
C23—H23	0.9300		
			
C6—N1—C2	110.7 (2)	C21—C22—H22	119.3
C6—N1—H11	111 (2)	C24—C23—C22	119.0 (3)
C2—N1—H11	108 (2)	C24—C23—H23	120.5
C6—N1—H12	106 (2)	C22—C23—H23	120.5
C2—N1—H12	113 (2)	C25—C24—C23	122.5 (3)
H11—N1—H12	108 (3)	C25—C24—F24	118.1 (4)
N1—C2—C3	111.1 (2)	C23—C24—F24	119.4 (4)
N1—C2—H2A	109.4	C24—C25—C26	119.2 (3)
C3—C2—H2A	109.4	C24—C25—H25	120.4
N1—C2—H2B	109.4	C26—C25—H25	120.4
C3—C2—H2B	109.4	C21—C26—C25	120.5 (3)
H2A—C2—H2B	108.0	C21—C26—H26	119.8
N4—C3—C2	110.3 (2)	C25—C26—H26	119.8
N4—C3—H3A	109.6	C36—C31—C32	117.6 (2)
C2—C3—H3A	109.6	C36—C31—C37	119.7 (2)
N4—C3—H3B	109.6	C32—C31—C37	122.8 (2)
C2—C3—H3B	109.6	C31—C32—C33	121.8 (3)
H3A—C3—H3B	108.1	C31—C32—Br32	120.83 (19)
C21—N4—C3	116.1 (2)	C33—C32—Br32	117.3 (2)
C21—N4—C5	114.7 (2)	C34—C33—C32	119.1 (3)
C3—N4—C5	109.0 (2)	C34—C33—H33	120.4
N4—C5—C6	111.5 (2)	C32—C33—H33	120.4
N4—C5—H5A	109.3	C33—C34—C35	120.4 (3)
C6—C5—H5A	109.3	C33—C34—H34	119.8
N4—C5—H5B	109.3	C35—C34—H34	119.8
C6—C5—H5B	109.3	C34—C35—C36	119.8 (3)
H5A—C5—H5B	108.0	C34—C35—H35	120.1
N1—C6—C5	110.8 (2)	C36—C35—H35	120.1
N1—C6—H6A	109.5	C31—C36—C35	121.2 (3)
C5—C6—H6A	109.5	C31—C36—H36	119.4
N1—C6—H6B	109.5	C35—C36—H36	119.4
C5—C6—H6B	109.5	O32—C37—O31	126.2 (3)
H6A—C6—H6B	108.1	O32—C37—C31	117.6 (2)
C26—C21—C22	117.5 (3)	O31—C37—C31	116.2 (3)
C26—C21—N4	119.2 (3)	O41^i^—O41—H41	88.0
C22—C21—N4	123.3 (2)	O41^i^—O41—H42	72.5
C23—C22—C21	121.3 (3)	H41—O41—H42	160.5
C23—C22—H22	119.3		
			
C6—N1—C2—C3	−54.9 (3)	C22—C21—C26—C25	1.1 (4)
N1—C2—C3—N4	58.5 (3)	N4—C21—C26—C25	179.9 (3)
C2—C3—N4—C21	168.8 (2)	C24—C25—C26—C21	−2.0 (5)
C2—C3—N4—C5	−59.8 (3)	C36—C31—C32—C33	−1.0 (4)
C21—N4—C5—C6	−168.4 (2)	C37—C31—C32—C33	178.4 (2)
C3—N4—C5—C6	59.5 (3)	C36—C31—C32—Br32	177.1 (2)
C2—N1—C6—C5	53.7 (3)	C37—C31—C32—Br32	−3.5 (3)
N4—C5—C6—N1	−56.6 (3)	C31—C32—C33—C34	−0.4 (4)
C3—N4—C21—C26	−171.3 (3)	Br32—C32—C33—C34	−178.5 (2)
C5—N4—C21—C26	60.0 (3)	C32—C33—C34—C35	1.4 (5)
C3—N4—C21—C22	7.4 (4)	C33—C34—C35—C36	−1.0 (5)
C5—N4—C21—C22	−121.2 (3)	C32—C31—C36—C35	1.3 (4)
C26—C21—C22—C23	0.4 (4)	C37—C31—C36—C35	−178.1 (3)
N4—C21—C22—C23	−178.3 (3)	C34—C35—C36—C31	−0.4 (5)
C21—C22—C23—C24	−1.1 (5)	C36—C31—C37—O32	111.7 (3)
C22—C23—C24—C25	0.2 (5)	C32—C31—C37—O32	−67.7 (3)
C22—C23—C24—F24	−178.5 (3)	C36—C31—C37—O31	−67.7 (4)
C23—C24—C25—C26	1.3 (6)	C32—C31—C37—O31	112.9 (3)
F24—C24—C25—C26	−180.0 (3)		

Symmetry code: (i) −*x*+1, −*y*, −*z*+1.

### 4-(4-Fluorophenyl)piperazin-1-ium 2-bromobenzoate 0.353-hydrate (II) Hydrogen-bond geometry (Å, º)

**Table:**

*D*—H···*A*	*D*—H	H···*A*	*D*···*A*	*D*—H···*A*
N1—H11···O32	0.78 (4)	1.93 (4)	2.677 (4)	160 (3)
N1—H12···O31^ii^	0.91 (3)	1.80 (3)	2.707 (4)	175 (3)
O41—H41···O31	0.90	1.76	2.661 (12)	179
O41—H42···O31^i^	0.89	1.90	2.792 (12)	179
C35—H35···O41^iii^	0.93	2.38	3.257 (11)	157
C2—H2*A*···*Cg*1^iv^	0.97	2.78	3.598 (3)	142

Symmetry codes: (i) −*x*+1, −*y*, −*z*+1; (ii) −*x*+1, −*y*+1, −*z*+1; (iii) *x*+1, *y*, *z*; (iv) *x*−1, *y*, *z*.

### 4-(4-Fluorophenyl)piperazin-1-ium 2-iodobenzoate (III) Crystal data

**Table:**

C_10_H_14_FN_2_^+^·C_7_H_4_IO_2_^−^	*Z* = 4
*M**_r_* = 428.23	*F*(000) = 848
Triclinic, *P*1	*D*_x_ = 1.634 Mg m^−^^3^
*a* = 9.8892 (3) Å	Mo *K*α radiation, λ = 0.71073 Å
*b* = 11.6831 (7) Å	Cell parameters from 7405 reflections
*c* = 16.4547 (9) Å	θ = 2.6–27.8°
α = 106.776 (5)°	µ = 1.86 mm^−^^1^
β = 93.327 (4)°	*T* = 293 K
γ = 104.874 (4)°	Block, orange
*V* = 1741.19 (16) Å^3^	0.50 × 0.50 × 0.48 mm

### 4-(4-Fluorophenyl)piperazin-1-ium 2-iodobenzoate (III) Data collection

**Table:**

Oxford Diffraction Xcalibur with Sapphire CCD diffractometer	7396 independent reflections
Radiation source: Enhance (Mo) X-ray Source	5890 reflections with *I* > 2σ(*I*)
Graphite monochromator	*R*_int_ = 0.015
ω scans	θ_max_ = 27.6°, θ_min_ = 2.6°
Absorption correction: multi-scan (CrysAlis RED; Oxford Diffraction, 2009)	*h* = −12→6
*T*_min_ = 0.383, *T*_max_ = 0.411	*k* = −14→15
12798 measured reflections	*l* = −21→20

### 4-(4-Fluorophenyl)piperazin-1-ium 2-iodobenzoate (III) Refinement

**Table:**

Refinement on *F*^2^	Primary atom site location: difference Fourier map
Least-squares matrix: full	Hydrogen site location: mixed
*R*[*F*^2^ > 2σ(*F*^2^)] = 0.047	H atoms treated by a mixture of independent and constrained refinement
*wR*(*F*^2^) = 0.127	*w* = 1/[σ^2^(*F*_o_^2^) + (0.0543*P*)^2^ + 4.1226*P*] where *P* = (*F*_o_^2^ + 2*F*_c_^2^)/3
*S* = 1.06	(Δ/σ)_max_ = 0.001
7396 reflections	Δρ_max_ = 2.92 e Å^−^^3^
427 parameters	Δρ_min_ = −2.00 e Å^−^^3^
0 restraints	

### 4-(4-Fluorophenyl)piperazin-1-ium 2-iodobenzoate (III) Special details

**Table:**

Geometry. All esds (except the esd in the dihedral angle between two l.s. planes) are estimated using the full covariance matrix. The cell esds are taken into account individually in the estimation of esds in distances, angles and torsion angles; correlations between esds in cell parameters are only used when they are defined by crystal symmetry. An approximate (isotropic) treatment of cell esds is used for estimating esds involving l.s. planes.

### 4-(4-Fluorophenyl)piperazin-1-ium 2-iodobenzoate (III) Fractional atomic coordinates and isotropic or equivalent isotropic displacement parameters (Å^2^)

**Table:**

	*x*	*y*	*z*	*U*_iso_*/*U*_eq_	
N11	0.1718 (4)	0.3441 (4)	0.4399 (3)	0.0473 (9)	
H111	0.168 (6)	0.371 (5)	0.490 (4)	0.057*	
H112	0.156 (6)	0.261 (5)	0.425 (3)	0.057*	
C12	0.3127 (5)	0.4007 (5)	0.4209 (3)	0.0592 (12)	
H12A	0.3831	0.3744	0.4486	0.071*	
H12B	0.3356	0.4909	0.4437	0.071*	
C13	0.3165 (5)	0.3626 (5)	0.3258 (3)	0.0546 (12)	
H13A	0.4093	0.4023	0.3146	0.066*	
H13B	0.3000	0.2730	0.3037	0.066*	
N14	0.2090 (4)	0.3984 (3)	0.2818 (3)	0.0450 (8)	
C15	0.0698 (5)	0.3408 (5)	0.2987 (4)	0.0579 (12)	
H15A	0.0483	0.2508	0.2752	0.069*	
H15B	−0.0002	0.3668	0.2704	0.069*	
C16	0.0617 (5)	0.3762 (5)	0.3932 (4)	0.0598 (13)	
H16A	0.0736	0.4652	0.4155	0.072*	
H16B	−0.0309	0.3330	0.4025	0.072*	
C121	0.2253 (5)	0.3925 (4)	0.1963 (3)	0.0487 (10)	
C122	0.3559 (6)	0.4465 (5)	0.1766 (4)	0.0597 (12)	
H122	0.4329	0.4829	0.2199	0.072*	
C123	0.3747 (7)	0.4476 (6)	0.0947 (4)	0.0741 (16)	
H123	0.4631	0.4837	0.0823	0.089*	
C124	0.2606 (9)	0.3945 (6)	0.0319 (4)	0.0760 (18)	
F124	0.2786 (6)	0.3960 (4)	−0.0494 (2)	0.1097 (15)	
C125	0.1323 (8)	0.3422 (6)	0.0477 (4)	0.0817 (19)	
H125	0.0563	0.3083	0.0037	0.098*	
C126	0.1126 (6)	0.3385 (5)	0.1294 (4)	0.0680 (15)	
H126	0.0238	0.2999	0.1400	0.082*	
N21	0.3249 (4)	0.1830 (4)	0.5932 (3)	0.0481 (9)	
H211	0.329 (6)	0.157 (5)	0.544 (4)	0.058*	
H212	0.337 (6)	0.261 (6)	0.611 (4)	0.058*	
C22	0.4376 (5)	0.1501 (6)	0.6362 (3)	0.0589 (13)	
H22A	0.5290	0.1980	0.6284	0.071*	
H22B	0.4291	0.0623	0.6100	0.071*	
C23	0.4291 (5)	0.1759 (5)	0.7303 (3)	0.0550 (12)	
H23A	0.5000	0.1479	0.7561	0.066*	
H23B	0.4487	0.2651	0.7578	0.066*	
N24	0.2907 (4)	0.1127 (3)	0.7441 (2)	0.0445 (8)	
C25	0.1819 (5)	0.1523 (5)	0.7055 (3)	0.0537 (11)	
H25A	0.1977	0.2412	0.7318	0.064*	
H25B	0.0899	0.1096	0.7160	0.064*	
C26	0.1841 (5)	0.1235 (5)	0.6102 (3)	0.0604 (13)	
H26A	0.1602	0.0340	0.5832	0.072*	
H26B	0.1141	0.1539	0.5859	0.072*	
C221	0.2749 (5)	0.0994 (4)	0.8257 (3)	0.0462 (10)	
C222	0.3844 (6)	0.1474 (5)	0.8936 (3)	0.0604 (13)	
H222	0.4722	0.1944	0.8871	0.072*	
C223	0.3638 (7)	0.1256 (6)	0.9712 (4)	0.0739 (16)	
H223	0.4373	0.1580	1.0167	0.089*	
C224	0.2374 (8)	0.0578 (6)	0.9801 (4)	0.0749 (17)	
F224	0.2184 (6)	0.0351 (5)	1.0561 (3)	0.1125 (15)	
C225	0.1270 (7)	0.0094 (6)	0.9158 (4)	0.0711 (15)	
H225	0.0400	−0.0366	0.9240	0.085*	
C226	0.1453 (5)	0.0295 (5)	0.8381 (4)	0.0573 (12)	
H226	0.0703	−0.0041	0.7935	0.069*	
C131	0.2741 (4)	0.5826 (4)	0.7440 (3)	0.0436 (9)	
C132	0.3185 (4)	0.7104 (4)	0.7577 (3)	0.0438 (9)	
I132	0.38641 (4)	0.78363 (3)	0.65990 (2)	0.06260 (12)	
C133	0.3235 (6)	0.7943 (5)	0.8376 (3)	0.0579 (12)	
H133	0.3542	0.8796	0.8461	0.069*	
C134	0.2831 (7)	0.7512 (6)	0.9039 (4)	0.0714 (16)	
H134	0.2850	0.8074	0.9574	0.086*	
C135	0.2401 (7)	0.6259 (6)	0.8920 (4)	0.0755 (17)	
H135	0.2128	0.5971	0.9374	0.091*	
C136	0.2371 (6)	0.5442 (5)	0.8148 (4)	0.0621 (13)	
H136	0.2097	0.4594	0.8083	0.075*	
C137	0.2666 (5)	0.4864 (4)	0.6585 (3)	0.0456 (10)	
O131	0.3594 (4)	0.4325 (3)	0.6506 (3)	0.0648 (10)	
O132	0.1659 (5)	0.4666 (4)	0.6041 (3)	0.0837 (13)	
C231	0.2056 (4)	−0.0826 (4)	0.3071 (3)	0.0408 (9)	
C232	0.1850 (4)	−0.1973 (4)	0.3215 (3)	0.0454 (10)	
I232	0.18832 (4)	−0.21282 (3)	0.44547 (2)	0.06302 (13)	
C233	0.1639 (6)	−0.3058 (5)	0.2536 (4)	0.0684 (15)	
H233	0.1502	−0.3819	0.2640	0.082*	
C234	0.1632 (8)	−0.3016 (6)	0.1721 (4)	0.0837 (19)	
H234	0.1488	−0.3748	0.1267	0.100*	
C235	0.1838 (7)	−0.1889 (6)	0.1561 (4)	0.0732 (16)	
H235	0.1839	−0.1862	0.1002	0.088*	
C236	0.2044 (5)	−0.0805 (5)	0.2232 (3)	0.0541 (11)	
H236	0.2175	−0.0050	0.2121	0.065*	
C237	0.2275 (5)	0.0386 (4)	0.3779 (3)	0.0434 (9)	
O231	0.1327 (4)	0.0902 (3)	0.3784 (3)	0.0689 (10)	
O232	0.3391 (4)	0.0769 (3)	0.4284 (2)	0.0619 (9)	

### 4-(4-Fluorophenyl)piperazin-1-ium 2-iodobenzoate (III) Atomic displacement parameters (Å^2^)

**Table:**

	*U*^11^	*U*^22^	*U*^33^	*U*^12^	*U*^13^	*U*^23^
N11	0.052 (2)	0.0355 (19)	0.053 (2)	0.0165 (17)	0.0012 (18)	0.0095 (17)
C12	0.042 (2)	0.063 (3)	0.063 (3)	0.006 (2)	−0.007 (2)	0.017 (2)
C13	0.034 (2)	0.065 (3)	0.068 (3)	0.016 (2)	−0.001 (2)	0.026 (3)
N14	0.0333 (17)	0.0402 (19)	0.062 (2)	0.0093 (14)	−0.0003 (16)	0.0192 (17)
C15	0.034 (2)	0.067 (3)	0.077 (3)	0.011 (2)	−0.003 (2)	0.033 (3)
C16	0.044 (3)	0.066 (3)	0.081 (4)	0.025 (2)	0.010 (2)	0.033 (3)
C121	0.047 (2)	0.034 (2)	0.067 (3)	0.0149 (18)	0.002 (2)	0.017 (2)
C122	0.055 (3)	0.056 (3)	0.063 (3)	0.010 (2)	0.005 (2)	0.018 (2)
C123	0.083 (4)	0.069 (4)	0.077 (4)	0.027 (3)	0.023 (3)	0.027 (3)
C124	0.122 (6)	0.061 (3)	0.056 (3)	0.042 (4)	0.013 (4)	0.021 (3)
F124	0.177 (5)	0.106 (3)	0.066 (2)	0.065 (3)	0.022 (3)	0.034 (2)
C125	0.095 (5)	0.071 (4)	0.069 (4)	0.023 (4)	−0.021 (4)	0.014 (3)
C126	0.058 (3)	0.065 (3)	0.071 (4)	0.007 (3)	−0.016 (3)	0.022 (3)
N21	0.053 (2)	0.043 (2)	0.048 (2)	0.0181 (18)	0.0033 (18)	0.0102 (18)
C22	0.045 (3)	0.079 (4)	0.068 (3)	0.030 (2)	0.015 (2)	0.033 (3)
C23	0.033 (2)	0.071 (3)	0.064 (3)	0.011 (2)	0.002 (2)	0.030 (3)
N24	0.0348 (18)	0.046 (2)	0.053 (2)	0.0125 (15)	0.0033 (15)	0.0151 (17)
C25	0.039 (2)	0.061 (3)	0.068 (3)	0.021 (2)	0.008 (2)	0.025 (2)
C26	0.045 (3)	0.070 (3)	0.064 (3)	0.014 (2)	−0.002 (2)	0.021 (3)
C221	0.047 (2)	0.039 (2)	0.054 (3)	0.0155 (19)	0.008 (2)	0.0128 (19)
C222	0.056 (3)	0.062 (3)	0.057 (3)	0.010 (2)	0.000 (2)	0.016 (2)
C223	0.084 (4)	0.080 (4)	0.057 (3)	0.027 (3)	0.000 (3)	0.019 (3)
C224	0.104 (5)	0.080 (4)	0.057 (3)	0.044 (4)	0.031 (3)	0.027 (3)
F224	0.148 (4)	0.148 (4)	0.075 (2)	0.065 (3)	0.044 (3)	0.061 (3)
C225	0.077 (4)	0.068 (4)	0.079 (4)	0.025 (3)	0.035 (3)	0.029 (3)
C226	0.051 (3)	0.054 (3)	0.067 (3)	0.014 (2)	0.015 (2)	0.018 (2)
C131	0.039 (2)	0.040 (2)	0.054 (2)	0.0174 (18)	0.0056 (18)	0.0126 (19)
C132	0.038 (2)	0.042 (2)	0.054 (2)	0.0167 (18)	0.0042 (18)	0.0143 (19)
I132	0.0718 (2)	0.0563 (2)	0.0754 (2)	0.02666 (17)	0.02048 (18)	0.03511 (18)
C133	0.062 (3)	0.043 (3)	0.063 (3)	0.021 (2)	0.004 (2)	0.003 (2)
C134	0.088 (4)	0.073 (4)	0.054 (3)	0.039 (3)	0.015 (3)	0.007 (3)
C135	0.097 (5)	0.083 (4)	0.067 (4)	0.044 (4)	0.033 (3)	0.035 (3)
C136	0.078 (4)	0.050 (3)	0.073 (3)	0.027 (3)	0.024 (3)	0.030 (3)
C137	0.040 (2)	0.033 (2)	0.060 (3)	0.0088 (17)	0.008 (2)	0.0102 (19)
O131	0.056 (2)	0.0489 (19)	0.082 (3)	0.0241 (16)	0.0081 (18)	0.0017 (18)
O132	0.074 (3)	0.097 (3)	0.063 (2)	0.043 (2)	−0.013 (2)	−0.013 (2)
C231	0.034 (2)	0.039 (2)	0.050 (2)	0.0118 (16)	0.0033 (17)	0.0134 (18)
C232	0.040 (2)	0.037 (2)	0.060 (3)	0.0146 (17)	0.0038 (19)	0.0125 (19)
I232	0.0707 (2)	0.0597 (2)	0.0752 (2)	0.02272 (17)	0.02547 (18)	0.03962 (18)
C233	0.079 (4)	0.041 (3)	0.079 (4)	0.021 (3)	0.006 (3)	0.006 (3)
C234	0.107 (5)	0.059 (4)	0.067 (4)	0.030 (3)	−0.003 (3)	−0.011 (3)
C235	0.091 (4)	0.079 (4)	0.046 (3)	0.035 (3)	0.001 (3)	0.007 (3)
C236	0.059 (3)	0.056 (3)	0.052 (3)	0.023 (2)	0.004 (2)	0.018 (2)
C237	0.050 (2)	0.033 (2)	0.047 (2)	0.0059 (18)	0.012 (2)	0.0170 (18)
O231	0.072 (2)	0.0392 (18)	0.090 (3)	0.0232 (17)	0.011 (2)	0.0065 (18)
O232	0.062 (2)	0.061 (2)	0.0474 (19)	0.0034 (17)	0.0019 (16)	0.0074 (16)

### 4-(4-Fluorophenyl)piperazin-1-ium 2-iodobenzoate (III) Geometric parameters (Å, º)

**Table:**

N11—C12	1.474 (6)	C25—H25B	0.9700
N11—C16	1.483 (6)	C26—H26A	0.9700
N11—H111	0.80 (6)	C26—H26B	0.9700
N11—H112	0.89 (6)	C221—C222	1.387 (7)
C12—C13	1.505 (7)	C221—C226	1.394 (7)
C12—H12A	0.9700	C222—C223	1.389 (8)
C12—H12B	0.9700	C222—H222	0.9300
C13—N14	1.463 (5)	C223—C224	1.338 (9)
C13—H13A	0.9700	C223—H223	0.9300
C13—H13B	0.9700	C224—C225	1.354 (9)
N14—C121	1.410 (6)	C224—F224	1.366 (7)
N14—C15	1.450 (6)	C225—C226	1.378 (8)
C15—C16	1.501 (8)	C225—H225	0.9300
C15—H15A	0.9700	C226—H226	0.9300
C15—H15B	0.9700	C131—C132	1.389 (6)
C16—H16A	0.9700	C131—C136	1.400 (7)
C16—H16B	0.9700	C131—C137	1.510 (6)
C121—C122	1.385 (7)	C132—C133	1.383 (7)
C121—C126	1.395 (7)	C132—I132	2.097 (5)
C122—C123	1.374 (8)	C133—C134	1.367 (8)
C122—H122	0.9300	C133—H133	0.9300
C123—C124	1.364 (10)	C134—C135	1.366 (9)
C123—H123	0.9300	C134—H134	0.9300
C124—C125	1.335 (10)	C135—C136	1.344 (8)
C124—F124	1.364 (7)	C135—H135	0.9300
C125—C126	1.381 (9)	C136—H136	0.9300
C125—H125	0.9300	C137—O132	1.228 (6)
C126—H126	0.9300	C137—O131	1.232 (5)
N21—C26	1.470 (7)	C231—C236	1.388 (7)
N21—C22	1.475 (6)	C231—C232	1.393 (6)
N21—H211	0.78 (6)	C231—C237	1.507 (6)
N21—H212	0.84 (6)	C232—C233	1.385 (7)
C22—C23	1.502 (7)	C232—I232	2.099 (5)
C22—H22A	0.9700	C233—C234	1.357 (9)
C22—H22B	0.9700	C233—H233	0.9300
C23—N24	1.444 (6)	C234—C235	1.383 (9)
C23—H23A	0.9700	C234—H234	0.9300
C23—H23B	0.9700	C235—C236	1.377 (7)
N24—C221	1.408 (6)	C235—H235	0.9300
N24—C25	1.456 (6)	C236—H236	0.9300
C25—C26	1.509 (7)	C237—O231	1.237 (6)
C25—H25A	0.9700	C237—O232	1.238 (6)
			
C12—N11—C16	110.2 (4)	N24—C25—C26	110.6 (4)
C12—N11—H111	110 (4)	N24—C25—H25A	109.5
C16—N11—H111	108 (4)	C26—C25—H25A	109.5
C12—N11—H112	109 (3)	N24—C25—H25B	109.5
C16—N11—H112	111 (3)	C26—C25—H25B	109.5
H111—N11—H112	109 (5)	H25A—C25—H25B	108.1
N11—C12—C13	110.8 (4)	N21—C26—C25	110.2 (4)
N11—C12—H12A	109.5	N21—C26—H26A	109.6
C13—C12—H12A	109.5	C25—C26—H26A	109.6
N11—C12—H12B	109.5	N21—C26—H26B	109.6
C13—C12—H12B	109.5	C25—C26—H26B	109.6
H12A—C12—H12B	108.1	H26A—C26—H26B	108.1
N14—C13—C12	110.8 (4)	C222—C221—C226	117.9 (5)
N14—C13—H13A	109.5	C222—C221—N24	123.2 (4)
C12—C13—H13A	109.5	C226—C221—N24	118.8 (4)
N14—C13—H13B	109.5	C221—C222—C223	120.3 (5)
C12—C13—H13B	109.5	C221—C222—H222	119.8
H13A—C13—H13B	108.1	C223—C222—H222	119.8
C121—N14—C15	117.6 (4)	C224—C223—C222	119.6 (6)
C121—N14—C13	114.6 (4)	C224—C223—H223	120.2
C15—N14—C13	110.1 (4)	C222—C223—H223	120.2
N14—C15—C16	111.3 (4)	C223—C224—C225	122.3 (6)
N14—C15—H15A	109.4	C223—C224—F224	119.3 (6)
C16—C15—H15A	109.4	C225—C224—F224	118.4 (6)
N14—C15—H15B	109.4	C224—C225—C226	119.1 (6)
C16—C15—H15B	109.4	C224—C225—H225	120.5
H15A—C15—H15B	108.0	C226—C225—H225	120.5
N11—C16—C15	111.3 (4)	C225—C226—C221	120.8 (5)
N11—C16—H16A	109.4	C225—C226—H226	119.6
C15—C16—H16A	109.4	C221—C226—H226	119.6
N11—C16—H16B	109.4	C132—C131—C136	116.7 (4)
C15—C16—H16B	109.4	C132—C131—C137	123.5 (4)
H16A—C16—H16B	108.0	C136—C131—C137	119.8 (4)
C122—C121—C126	117.4 (5)	C133—C132—C131	120.9 (4)
C122—C121—N14	120.0 (4)	C133—C132—I132	117.5 (4)
C126—C121—N14	122.6 (5)	C131—C132—I132	121.6 (3)
C123—C122—C121	121.7 (5)	C134—C133—C132	119.7 (5)
C123—C122—H122	119.1	C134—C133—H133	120.1
C121—C122—H122	119.1	C132—C133—H133	120.1
C124—C123—C122	118.5 (6)	C135—C134—C133	120.3 (5)
C124—C123—H123	120.8	C135—C134—H134	119.8
C122—C123—H123	120.8	C133—C134—H134	119.8
C125—C124—C123	122.1 (6)	C136—C135—C134	120.1 (6)
C125—C124—F124	119.2 (7)	C136—C135—H135	120.0
C123—C124—F124	118.7 (7)	C134—C135—H135	120.0
C124—C125—C126	120.0 (6)	C135—C136—C131	122.2 (5)
C124—C125—H125	120.0	C135—C136—H136	118.9
C126—C125—H125	120.0	C131—C136—H136	118.9
C125—C126—C121	120.3 (6)	O132—C137—O131	126.0 (5)
C125—C126—H126	119.9	O132—C137—C131	116.7 (4)
C121—C126—H126	119.9	O131—C137—C131	117.3 (4)
C26—N21—C22	112.1 (4)	C236—C231—C232	118.2 (4)
C26—N21—H211	109 (4)	C236—C231—C237	118.5 (4)
C22—N21—H211	105 (4)	C232—C231—C237	123.3 (4)
C26—N21—H212	107 (4)	C233—C232—C231	120.7 (5)
C22—N21—H212	110 (4)	C233—C232—I232	117.4 (4)
H211—N21—H212	114 (6)	C231—C232—I232	121.9 (3)
N21—C22—C23	111.3 (4)	C234—C233—C232	120.2 (5)
N21—C22—H22A	109.4	C234—C233—H233	119.9
C23—C22—H22A	109.4	C232—C233—H233	119.9
N21—C22—H22B	109.4	C233—C234—C235	120.3 (5)
C23—C22—H22B	109.4	C233—C234—H234	119.9
H22A—C22—H22B	108.0	C235—C234—H234	119.9
N24—C23—C22	110.7 (4)	C236—C235—C234	119.9 (5)
N24—C23—H23A	109.5	C236—C235—H235	120.0
C22—C23—H23A	109.5	C234—C235—H235	120.0
N24—C23—H23B	109.5	C235—C236—C231	120.8 (5)
C22—C23—H23B	109.5	C235—C236—H236	119.6
H23A—C23—H23B	108.1	C231—C236—H236	119.6
C221—N24—C23	118.0 (4)	O231—C237—O232	127.0 (4)
C221—N24—C25	116.3 (4)	O231—C237—C231	116.2 (4)
C23—N24—C25	110.7 (4)	O232—C237—C231	116.7 (4)
			
C16—N11—C12—C13	−55.3 (6)	C222—C223—C224—C225	−0.4 (10)
N11—C12—C13—N14	57.8 (6)	C222—C223—C224—F224	179.0 (5)
C12—C13—N14—C121	166.2 (4)	C223—C224—C225—C226	0.7 (10)
C12—C13—N14—C15	−58.5 (5)	F224—C224—C225—C226	−178.7 (5)
C121—N14—C15—C16	−168.6 (4)	C224—C225—C226—C221	−0.6 (8)
C13—N14—C15—C16	57.6 (5)	C222—C221—C226—C225	0.3 (7)
C12—N11—C16—C15	54.5 (6)	N24—C221—C226—C225	177.1 (5)
N14—C15—C16—N11	−56.1 (6)	C136—C131—C132—C133	−0.9 (6)
C15—N14—C121—C122	179.7 (4)	C137—C131—C132—C133	−179.8 (4)
C13—N14—C121—C122	−48.5 (6)	C136—C131—C132—I132	177.2 (3)
C15—N14—C121—C126	2.6 (6)	C137—C131—C132—I132	−1.8 (6)
C13—N14—C121—C126	134.4 (5)	C131—C132—C133—C134	−0.5 (7)
C126—C121—C122—C123	0.2 (8)	I132—C132—C133—C134	−178.6 (4)
N14—C121—C122—C123	−177.1 (5)	C132—C133—C134—C135	1.0 (9)
C121—C122—C123—C124	0.4 (9)	C133—C134—C135—C136	0.0 (10)
C122—C123—C124—C125	0.2 (10)	C134—C135—C136—C131	−1.5 (10)
C122—C123—C124—F124	179.6 (5)	C132—C131—C136—C135	1.9 (8)
C123—C124—C125—C126	−1.3 (10)	C137—C131—C136—C135	−179.1 (5)
F124—C124—C125—C126	179.2 (5)	C132—C131—C137—O132	−77.1 (6)
C124—C125—C126—C121	1.9 (10)	C136—C131—C137—O132	103.9 (6)
C122—C121—C126—C125	−1.3 (8)	C132—C131—C137—O131	104.8 (5)
N14—C121—C126—C125	175.9 (5)	C136—C131—C137—O131	−74.1 (6)
C26—N21—C22—C23	−53.0 (6)	C236—C231—C232—C233	0.0 (7)
N21—C22—C23—N24	55.1 (6)	C237—C231—C232—C233	179.3 (5)
C22—C23—N24—C221	163.7 (4)	C236—C231—C232—I232	178.8 (3)
C22—C23—N24—C25	−58.7 (5)	C237—C231—C232—I232	−1.8 (6)
C221—N24—C25—C26	−161.9 (4)	C231—C232—C233—C234	0.1 (8)
C23—N24—C25—C26	59.8 (5)	I232—C232—C233—C234	−178.8 (5)
C22—N21—C26—C25	53.6 (6)	C232—C233—C234—C235	0.1 (10)
N24—C25—C26—N21	−56.6 (6)	C233—C234—C235—C236	−0.4 (10)
C23—N24—C221—C222	2.1 (7)	C234—C235—C236—C231	0.4 (9)
C25—N24—C221—C222	−133.2 (5)	C232—C231—C236—C235	−0.2 (7)
C23—N24—C221—C226	−174.5 (4)	C237—C231—C236—C235	−179.6 (5)
C25—N24—C221—C226	50.2 (6)	C236—C231—C237—O231	66.0 (6)
C226—C221—C222—C223	−0.1 (8)	C232—C231—C237—O231	−113.4 (5)
N24—C221—C222—C223	−176.7 (5)	C236—C231—C237—O232	−112.9 (5)
C221—C222—C223—C224	0.1 (9)	C232—C231—C237—O232	67.8 (6)

### 4-(4-Fluorophenyl)piperazin-1-ium 2-iodobenzoate (III) Hydrogen-bond geometry (Å, º)

**Table:**

*D*—H···*A*	*D*—H	H···*A*	*D*···*A*	*D*—H···*A*
N11—H111···O132	0.80 (6)	1.89 (6)	2.680 (7)	168 (6)
N11—H112···O231	0.90 (6)	1.87 (6)	2.758 (6)	171 (4)
N21—H211···O232	0.79 (6)	1.88 (6)	2.665 (6)	173 (6)
N21—H212···O131	0.85 (7)	1.87 (7)	2.714 (6)	179 (8)
C13—H13*A*···O131^i^	0.97	2.50	3.396 (7)	154
C133—H133···*Cg*2^ii^	0.93	2.69	3.433 (6)	137

Symmetry codes: (i) −*x*+1, −*y*+1, −*z*+1; (ii) *x*, *y*+1, *z*.

### 4-(4-Fluorophenyl)piperazin-1-ium 2,4,6-trinitrophenolate (IV) Crystal data

**Table:**

C_10_H_14_FN_2_^+^·C_6_H_2_N_3_O_7_^−^	*F*(000) = 848
*M**_r_* = 409.34	*D*_x_ = 1.576 Mg m^−^^3^
Monoclinic, *P*2_1_/*n*	Mo *K*α radiation, λ = 0.71073 Å
*a* = 16.658 (2) Å	Cell parameters from 3865 reflections
*b* = 6.6734 (6) Å	θ = 2.6–27.8°
*c* = 17.553 (3) Å	µ = 0.13 mm^−^^1^
β = 117.84 (2)°	*T* = 293 K
*V* = 1725.4 (5) Å^3^	Block, orange
*Z* = 4	0.48 × 0.40 × 0.40 mm

### 4-(4-Fluorophenyl)piperazin-1-ium 2,4,6-trinitrophenolate (IV) Data collection

**Table:**

Oxford Diffraction Xcalibur with Sapphire CCD diffractometer	3865 independent reflections
Radiation source: Enhance (Mo) X-ray Source	2922 reflections with *I* > 2σ(*I*)
Graphite monochromator	*R*_int_ = 0.019
ω scans	θ_max_ = 27.8°, θ_min_ = 2.6°
Absorption correction: multi-scan (CrysAlis RED; Oxford Diffraction, 2009)	*h* = −21→20
*T*_min_ = 0.816, *T*_max_ = 0.949	*k* = −8→8
12153 measured reflections	*l* = −23→17

### 4-(4-Fluorophenyl)piperazin-1-ium 2,4,6-trinitrophenolate (IV) Refinement

**Table:**

Refinement on *F*^2^	Hydrogen site location: mixed
Least-squares matrix: full	H atoms treated by a mixture of independent and constrained refinement
*R*[*F*^2^ > 2σ(*F*^2^)] = 0.045	*w* = 1/[σ^2^(*F*_o_^2^) + (0.069*P*)^2^ + 0.535*P*] where *P* = (*F*_o_^2^ + 2*F*_c_^2^)/3
*wR*(*F*^2^) = 0.136	(Δ/σ)_max_ = 0.001
*S* = 1.05	Δρ_max_ = 0.45 e Å^−^^3^
3865 reflections	Δρ_min_ = −0.27 e Å^−^^3^
269 parameters	Extinction correction: SHELXL, Fc^*^=kFc[1+0.001xFc^2^λ^3^/sin(2θ)]^-1/4^
0 restraints	Extinction coefficient: 0.0063 (12)
Primary atom site location: difference Fourier map	

### 4-(4-Fluorophenyl)piperazin-1-ium 2,4,6-trinitrophenolate (IV) Special details

**Table:**

Geometry. All esds (except the esd in the dihedral angle between two l.s. planes) are estimated using the full covariance matrix. The cell esds are taken into account individually in the estimation of esds in distances, angles and torsion angles; correlations between esds in cell parameters are only used when they are defined by crystal symmetry. An approximate (isotropic) treatment of cell esds is used for estimating esds involving l.s. planes.

### 4-(4-Fluorophenyl)piperazin-1-ium 2,4,6-trinitrophenolate (IV) Fractional atomic coordinates and isotropic or equivalent isotropic displacement parameters (Å^2^)

**Table:**

	*x*	*y*	*z*	*U*_iso_*/*U*_eq_	
N1	0.40587 (10)	0.4532 (3)	0.63092 (10)	0.0437 (4)	
H11	0.4673 (15)	0.458 (3)	0.6543 (14)	0.052*	
H12	0.3839 (14)	0.402 (3)	0.5813 (15)	0.052*	
C2	0.37290 (12)	0.6586 (3)	0.62596 (14)	0.0539 (5)	
H2A	0.3861	0.7353	0.5862	0.065*	
H2B	0.4045	0.7211	0.6822	0.065*	
C3	0.27242 (12)	0.6622 (3)	0.59632 (15)	0.0562 (5)	
H3A	0.2532	0.7996	0.5958	0.067*	
H3B	0.2408	0.6114	0.5378	0.067*	
N4	0.24731 (9)	0.5429 (2)	0.65119 (9)	0.0368 (3)	
C5	0.27922 (12)	0.3385 (3)	0.65583 (15)	0.0554 (5)	
H5A	0.2471	0.2767	0.5995	0.066*	
H5B	0.2656	0.2626	0.6955	0.066*	
C6	0.37933 (13)	0.3300 (4)	0.68511 (16)	0.0659 (6)	
H6A	0.4117	0.3765	0.7442	0.079*	
H6B	0.3969	0.1921	0.6837	0.079*	
C21	0.15488 (10)	0.5597 (3)	0.63263 (10)	0.0363 (4)	
C22	0.10464 (13)	0.7296 (3)	0.59483 (14)	0.0564 (5)	
H22	0.1312	0.8344	0.5798	0.068*	
C23	0.01531 (13)	0.7464 (4)	0.57899 (14)	0.0654 (6)	
H23	−0.0176	0.8621	0.5539	0.078*	
C24	−0.02378 (12)	0.5940 (4)	0.60014 (12)	0.0533 (5)	
F24	−0.11190 (7)	0.6095 (3)	0.58299 (9)	0.0781 (4)	
C25	0.02295 (13)	0.4251 (3)	0.63800 (15)	0.0582 (5)	
H25	−0.0048	0.3218	0.6526	0.070*	
C26	0.11240 (12)	0.4082 (3)	0.65464 (14)	0.0530 (5)	
H26	0.1448	0.2929	0.6811	0.064*	
C31	0.66352 (10)	0.5012 (2)	0.76514 (10)	0.0325 (3)	
O31	0.58276 (8)	0.4723 (2)	0.74598 (8)	0.0515 (4)	
C32	0.69912 (10)	0.5164 (2)	0.70408 (9)	0.0311 (3)	
C33	0.78970 (10)	0.5187 (2)	0.72642 (10)	0.0316 (3)	
H33	0.8087	0.5195	0.6843	0.038*	
C34	0.85232 (10)	0.5198 (2)	0.81288 (10)	0.0310 (3)	
C35	0.82562 (10)	0.5169 (2)	0.87651 (10)	0.0307 (3)	
H35	0.8686	0.5201	0.9343	0.037*	
C36	0.73523 (10)	0.5092 (2)	0.85326 (9)	0.0306 (3)	
N32	0.63656 (10)	0.5325 (2)	0.61270 (9)	0.0408 (3)	
O32	0.55940 (9)	0.5932 (3)	0.59024 (9)	0.0663 (4)	
O33	0.66548 (10)	0.4937 (3)	0.56166 (9)	0.0652 (4)	
N34	0.94736 (9)	0.5289 (2)	0.83657 (10)	0.0408 (3)	
O34	0.97031 (9)	0.4935 (2)	0.78121 (10)	0.0596 (4)	
O35	1.00128 (8)	0.5720 (3)	0.91068 (9)	0.0653 (4)	
N36	0.71188 (10)	0.5052 (2)	0.92360 (9)	0.0415 (3)	
O36	0.76681 (10)	0.4316 (2)	0.99197 (8)	0.0586 (4)	
O37	0.64114 (10)	0.5790 (3)	0.91295 (10)	0.0662 (4)	

### 4-(4-Fluorophenyl)piperazin-1-ium 2,4,6-trinitrophenolate (IV) Atomic displacement parameters (Å^2^)

**Table:**

	*U*^11^	*U*^22^	*U*^33^	*U*^12^	*U*^13^	*U*^23^
N1	0.0255 (7)	0.0670 (10)	0.0364 (7)	0.0038 (7)	0.0125 (6)	−0.0009 (7)
C2	0.0398 (9)	0.0577 (12)	0.0692 (13)	−0.0043 (9)	0.0296 (9)	0.0047 (10)
C3	0.0405 (10)	0.0588 (12)	0.0768 (14)	0.0120 (9)	0.0336 (10)	0.0262 (10)
N4	0.0308 (7)	0.0434 (8)	0.0381 (7)	0.0044 (6)	0.0178 (6)	0.0026 (6)
C5	0.0435 (10)	0.0489 (11)	0.0845 (14)	0.0110 (8)	0.0389 (10)	0.0155 (10)
C6	0.0454 (11)	0.0752 (15)	0.0866 (15)	0.0254 (10)	0.0389 (11)	0.0360 (13)
C21	0.0310 (8)	0.0481 (9)	0.0304 (7)	0.0037 (7)	0.0150 (6)	−0.0019 (7)
C22	0.0460 (10)	0.0672 (13)	0.0647 (12)	0.0181 (9)	0.0330 (9)	0.0228 (10)
C23	0.0470 (11)	0.0883 (17)	0.0643 (13)	0.0309 (11)	0.0289 (10)	0.0238 (12)
C24	0.0305 (8)	0.0867 (15)	0.0421 (10)	0.0051 (9)	0.0165 (8)	−0.0072 (10)
F24	0.0314 (6)	0.1237 (12)	0.0776 (9)	0.0088 (7)	0.0241 (6)	−0.0065 (8)
C25	0.0423 (10)	0.0666 (13)	0.0737 (14)	−0.0085 (9)	0.0337 (10)	−0.0089 (11)
C26	0.0417 (10)	0.0512 (11)	0.0719 (13)	0.0038 (8)	0.0314 (10)	0.0038 (10)
C31	0.0254 (7)	0.0299 (7)	0.0367 (8)	0.0004 (6)	0.0099 (6)	−0.0011 (6)
O31	0.0251 (6)	0.0758 (9)	0.0483 (7)	−0.0046 (6)	0.0125 (5)	0.0002 (6)
C32	0.0301 (7)	0.0278 (7)	0.0273 (7)	−0.0015 (6)	0.0066 (6)	−0.0006 (6)
C33	0.0349 (8)	0.0283 (7)	0.0331 (7)	−0.0013 (6)	0.0171 (6)	0.0003 (6)
C34	0.0241 (7)	0.0294 (7)	0.0366 (8)	−0.0002 (6)	0.0119 (6)	0.0013 (6)
C35	0.0276 (7)	0.0290 (7)	0.0292 (7)	−0.0008 (6)	0.0081 (6)	−0.0008 (6)
C36	0.0295 (7)	0.0308 (7)	0.0307 (7)	−0.0008 (6)	0.0135 (6)	−0.0021 (6)
N32	0.0405 (8)	0.0403 (8)	0.0305 (7)	−0.0064 (6)	0.0073 (6)	−0.0005 (6)
O32	0.0406 (7)	0.0907 (12)	0.0437 (8)	0.0081 (7)	−0.0003 (6)	0.0033 (7)
O33	0.0647 (9)	0.0950 (12)	0.0317 (7)	0.0007 (8)	0.0190 (6)	0.0027 (7)
N34	0.0274 (7)	0.0446 (8)	0.0493 (8)	0.0015 (6)	0.0169 (6)	0.0076 (6)
O34	0.0411 (7)	0.0796 (10)	0.0699 (9)	0.0047 (7)	0.0358 (7)	0.0033 (8)
O35	0.0281 (6)	0.1017 (12)	0.0520 (8)	−0.0113 (7)	0.0069 (6)	−0.0006 (8)
N36	0.0383 (7)	0.0483 (8)	0.0417 (8)	−0.0061 (6)	0.0219 (6)	−0.0090 (6)
O36	0.0569 (8)	0.0831 (11)	0.0368 (7)	0.0005 (7)	0.0226 (6)	0.0068 (7)
O37	0.0517 (8)	0.0930 (12)	0.0654 (9)	0.0102 (8)	0.0369 (7)	−0.0124 (8)

### 4-(4-Fluorophenyl)piperazin-1-ium 2,4,6-trinitrophenolate (IV) Geometric parameters (Å, º)

**Table:**

N1—C2	1.464 (3)	C24—C25	1.355 (3)
N1—C6	1.473 (3)	C24—F24	1.358 (2)
N1—H11	0.91 (2)	C25—C26	1.383 (2)
N1—H12	0.84 (2)	C25—H25	0.9300
C2—C3	1.503 (2)	C26—H26	0.9300
C2—H2A	0.9700	C31—O31	1.2398 (19)
C2—H2B	0.9700	C31—C36	1.448 (2)
C3—N4	1.453 (2)	C31—C32	1.451 (2)
C3—H3A	0.9700	C32—C33	1.371 (2)
C3—H3B	0.9700	C32—N32	1.4522 (19)
N4—C21	1.4215 (19)	C33—C34	1.383 (2)
N4—C5	1.452 (2)	C33—H33	0.9300
C5—C6	1.499 (2)	C34—C35	1.382 (2)
C5—H5A	0.9700	C34—N34	1.4383 (19)
C5—H5B	0.9700	C35—C36	1.365 (2)
C6—H6A	0.9700	C35—H35	0.9300
C6—H6B	0.9700	C36—N36	1.458 (2)
C21—C22	1.381 (3)	N32—O32	1.224 (2)
C21—C26	1.388 (3)	N32—O33	1.227 (2)
C22—C23	1.385 (3)	N34—O35	1.221 (2)
C22—H22	0.9300	N34—O34	1.222 (2)
C23—C24	1.350 (3)	N36—O37	1.2084 (19)
C23—H23	0.9300	N36—O36	1.222 (2)
			
C2—N1—C6	110.09 (15)	C24—C23—C22	119.7 (2)
C2—N1—H11	107.8 (13)	C24—C23—H23	120.2
C6—N1—H11	110.0 (13)	C22—C23—H23	120.2
C2—N1—H12	110.5 (15)	C23—C24—C25	121.43 (17)
C6—N1—H12	108.8 (15)	C23—C24—F24	119.5 (2)
H11—N1—H12	109.7 (19)	C25—C24—F24	119.1 (2)
N1—C2—C3	111.20 (16)	C24—C25—C26	119.2 (2)
N1—C2—H2A	109.4	C24—C25—H25	120.4
C3—C2—H2A	109.4	C26—C25—H25	120.4
N1—C2—H2B	109.4	C25—C26—C21	121.39 (19)
C3—C2—H2B	109.4	C25—C26—H26	119.3
H2A—C2—H2B	108.0	C21—C26—H26	119.3
N4—C3—C2	112.37 (16)	O31—C31—C36	123.02 (15)
N4—C3—H3A	109.1	O31—C31—C32	125.20 (15)
C2—C3—H3A	109.1	C36—C31—C32	111.63 (13)
N4—C3—H3B	109.1	C33—C32—C31	124.38 (14)
C2—C3—H3B	109.1	C33—C32—N32	116.20 (14)
H3A—C3—H3B	107.9	C31—C32—N32	119.41 (14)
C21—N4—C5	114.59 (14)	C32—C33—C34	118.65 (14)
C21—N4—C3	114.90 (13)	C32—C33—H33	120.7
C5—N4—C3	109.90 (14)	C34—C33—H33	120.7
N4—C5—C6	111.98 (17)	C35—C34—C33	121.63 (14)
N4—C5—H5A	109.2	C35—C34—N34	119.56 (14)
C6—C5—H5A	109.2	C33—C34—N34	118.80 (14)
N4—C5—H5B	109.2	C36—C35—C34	119.04 (14)
C6—C5—H5B	109.2	C36—C35—H35	120.5
H5A—C5—H5B	107.9	C34—C35—H35	120.5
N1—C6—C5	112.26 (16)	C35—C36—C31	124.43 (14)
N1—C6—H6A	109.2	C35—C36—N36	116.17 (13)
C5—C6—H6A	109.2	C31—C36—N36	119.39 (13)
N1—C6—H6B	109.2	O32—N32—O33	123.09 (15)
C5—C6—H6B	109.2	O32—N32—C32	118.97 (15)
H6A—C6—H6B	107.9	O33—N32—C32	117.84 (15)
C22—C21—C26	117.27 (16)	O35—N34—O34	123.04 (15)
C22—C21—N4	121.57 (16)	O35—N34—C34	118.74 (15)
C26—C21—N4	121.12 (15)	O34—N34—C34	118.22 (15)
C21—C22—C23	121.1 (2)	O37—N36—O36	123.00 (15)
C21—C22—H22	119.5	O37—N36—C36	119.22 (15)
C23—C22—H22	119.5	O36—N36—C36	117.74 (14)
			
C6—N1—C2—C3	−54.1 (2)	C36—C31—C32—N32	−173.50 (13)
N1—C2—C3—N4	56.7 (2)	C31—C32—C33—C34	−4.4 (2)
C2—C3—N4—C21	172.77 (16)	N32—C32—C33—C34	174.93 (13)
C2—C3—N4—C5	−56.2 (2)	C32—C33—C34—C35	0.6 (2)
C21—N4—C5—C6	−173.67 (16)	C32—C33—C34—N34	−177.97 (14)
C3—N4—C5—C6	55.2 (2)	C33—C34—C35—C36	1.2 (2)
C2—N1—C6—C5	53.9 (3)	N34—C34—C35—C36	179.69 (14)
N4—C5—C6—N1	−55.2 (3)	C34—C35—C36—C31	0.8 (2)
C5—N4—C21—C22	−155.31 (19)	C34—C35—C36—N36	179.64 (14)
C3—N4—C21—C22	−26.6 (2)	O31—C31—C36—C35	171.87 (16)
C5—N4—C21—C26	27.0 (2)	C32—C31—C36—C35	−4.0 (2)
C3—N4—C21—C26	155.66 (19)	O31—C31—C36—N36	−6.9 (2)
C26—C21—C22—C23	−0.6 (3)	C32—C31—C36—N36	177.23 (13)
N4—C21—C22—C23	−178.38 (18)	C33—C32—N32—O32	−157.44 (16)
C21—C22—C23—C24	−0.5 (3)	C31—C32—N32—O32	21.9 (2)
C22—C23—C24—C25	1.1 (3)	C33—C32—N32—O33	19.0 (2)
C22—C23—C24—F24	−178.73 (19)	C31—C32—N32—O33	−161.62 (16)
C23—C24—C25—C26	−0.5 (3)	C35—C34—N34—O35	−13.8 (2)
F24—C24—C25—C26	179.29 (18)	C33—C34—N34—O35	164.79 (16)
C24—C25—C26—C21	−0.6 (3)	C35—C34—N34—O34	166.42 (15)
C22—C21—C26—C25	1.1 (3)	C33—C34—N34—O34	−15.0 (2)
N4—C21—C26—C25	178.95 (18)	C35—C36—N36—O37	149.59 (16)
O31—C31—C32—C33	−169.91 (16)	C31—C36—N36—O37	−31.5 (2)
C36—C31—C32—C33	5.8 (2)	C35—C36—N36—O36	−28.3 (2)
O31—C31—C32—N32	10.8 (2)	C31—C36—N36—O36	150.60 (16)

### 4-(4-Fluorophenyl)piperazin-1-ium 2,4,6-trinitrophenolate (IV) Hydrogen-bond geometry (Å, º)

**Table:**

*D*—H···*A*	*D*—H	H···*A*	*D*···*A*	*D*—H···*A*
N1—H11···O31	0.91 (3)	1.85 (2)	2.687 (2)	153 (2)
N1—H11···O32	0.91 (3)	2.46 (3)	3.103 (3)	128.2 (17)
N1—H12···O33^i^	0.84 (2)	2.35 (2)	3.035 (2)	138.5 (18)
C5—H5*A*···O36^ii^	0.97	2.49	3.318 (3)	144
C6—H6*B*···O34^iii^	0.97	2.40	3.207 (3)	140
C25—H25···O37^iv^	0.93	2.58	3.365 (3)	142

Symmetry codes: (i) −*x*+1, −*y*+1, −*z*+1; (ii) *x*−1/2, −*y*+1/2, *z*−1/2; (iii) −*x*+3/2, *y*−1/2, −*z*+3/2; (iv) −*x*+1/2, *y*−1/2, −*z*+3/2.

### 4-(4-Fluorophenyl)piperazin-1-ium 3,5-dinitrobenzoate (V) Crystal data

**Table:**

C_10_H_14_FN_2_^+^·C_7_H_3_N_2_O_6_^−^	*F*(000) = 1632
*M**_r_* = 392.34	*D*_x_ = 1.362 Mg m^−^^3^
Monoclinic, *C*2/*c*	Mo *K*α radiation, λ = 0.71073 Å
*a* = 19.871 (1) Å	Cell parameters from 4082 reflections
*b* = 7.3420 (7) Å	θ = 3.0–27.7°
*c* = 26.306 (2) Å	µ = 0.11 mm^−^^1^
β = 94.540 (8)°	*T* = 293 K
*V* = 3825.8 (5) Å^3^	Plate, orange
*Z* = 8	0.46 × 0.42 × 0.22 mm

### 4-(4-Fluorophenyl)piperazin-1-ium 3,5-dinitrobenzoate (V) Data collection

**Table:**

Oxford Diffraction Xcalibur with Sapphire CCD diffractometer	4080 independent reflections
Radiation source: Enhance (Mo) X-ray Source	2490 reflections with *I* > 2σ(*I*)
Graphite monochromator	*R*_int_ = 0.017
ω scans	θ_max_ = 27.7°, θ_min_ = 3.0°
Absorption correction: multi-scan (CrysAlis RED; Oxford Diffraction, 2009)	*h* = −26→20
*T*_min_ = 0.832, *T*_max_ = 0.976	*k* = −7→9
7938 measured reflections	*l* = −34→29

### 4-(4-Fluorophenyl)piperazin-1-ium 3,5-dinitrobenzoate (V) Refinement

**Table:**

Refinement on *F*^2^	Primary atom site location: difference Fourier map
Least-squares matrix: full	Hydrogen site location: mixed
*R*[*F*^2^ > 2σ(*F*^2^)] = 0.056	H atoms treated by a mixture of independent and constrained refinement
*wR*(*F*^2^) = 0.144	*w* = 1/[σ^2^(*F*_o_^2^) + (0.0501*P*)^2^ + 3.0431*P*] where *P* = (*F*_o_^2^ + 2*F*_c_^2^)/3
*S* = 1.07	(Δ/σ)_max_ < 0.001
4080 reflections	Δρ_max_ = 0.19 e Å^−^^3^
259 parameters	Δρ_min_ = −0.18 e Å^−^^3^
0 restraints	

### 4-(4-Fluorophenyl)piperazin-1-ium 3,5-dinitrobenzoate (V) Special details

**Table:**

Geometry. All esds (except the esd in the dihedral angle between two l.s. planes) are estimated using the full covariance matrix. The cell esds are taken into account individually in the estimation of esds in distances, angles and torsion angles; correlations between esds in cell parameters are only used when they are defined by crystal symmetry. An approximate (isotropic) treatment of cell esds is used for estimating esds involving l.s. planes.

### 4-(4-Fluorophenyl)piperazin-1-ium 3,5-dinitrobenzoate (V) Fractional atomic coordinates and isotropic or equivalent isotropic displacement parameters (Å^2^)

**Table:**

	*x*	*y*	*z*	*U*_iso_*/*U*_eq_	
N1	0.50399 (11)	0.4508 (4)	0.58562 (8)	0.0639 (7)	
H11	0.4607 (15)	0.472 (4)	0.5700 (10)	0.077*	
H12	0.5386 (14)	0.469 (4)	0.5632 (10)	0.077*	
C2	0.50591 (13)	0.2591 (4)	0.60334 (10)	0.0693 (8)	
H2A	0.5042	0.1785	0.5741	0.083*	
H2B	0.4666	0.2348	0.6219	0.083*	
C3	0.56927 (12)	0.2204 (4)	0.63747 (9)	0.0605 (7)	
H3A	0.5685	0.0957	0.6496	0.073*	
H3B	0.6087	0.2357	0.6184	0.073*	
N4	0.57304 (9)	0.3446 (3)	0.68058 (7)	0.0499 (5)	
C5	0.57665 (12)	0.5329 (4)	0.66245 (9)	0.0578 (6)	
H5A	0.6160	0.5475	0.6433	0.069*	
H5B	0.5811	0.6152	0.6914	0.069*	
C6	0.51379 (12)	0.5795 (4)	0.62894 (9)	0.0601 (7)	
H6A	0.4749	0.5748	0.6490	0.072*	
H6B	0.5175	0.7026	0.6160	0.072*	
C21	0.61998 (10)	0.3004 (3)	0.72265 (8)	0.0474 (6)	
C22	0.66827 (12)	0.1663 (4)	0.72083 (9)	0.0603 (7)	
H22	0.6724	0.1034	0.6906	0.072*	
C23	0.71091 (13)	0.1240 (4)	0.76380 (10)	0.0697 (8)	
H23	0.7429	0.0319	0.7627	0.084*	
C24	0.70484 (13)	0.2199 (4)	0.80725 (10)	0.0616 (7)	
F24	0.74665 (9)	0.1803 (3)	0.84952 (6)	0.0940 (6)	
C25	0.65828 (14)	0.3538 (4)	0.81063 (9)	0.0649 (7)	
H25	0.6553	0.4173	0.8409	0.078*	
C26	0.61553 (12)	0.3937 (4)	0.76819 (9)	0.0596 (7)	
H26	0.5832	0.4845	0.7701	0.072*	
C31	0.28091 (10)	0.5173 (3)	0.49463 (8)	0.0426 (5)	
C32	0.25827 (11)	0.5776 (3)	0.44636 (9)	0.0488 (6)	
H32	0.2890	0.6164	0.4237	0.059*	
C33	0.19032 (12)	0.5798 (3)	0.43210 (8)	0.0504 (6)	
C34	0.14305 (12)	0.5225 (3)	0.46380 (9)	0.0522 (6)	
H34	0.0972	0.5242	0.4536	0.063*	
C35	0.16663 (11)	0.4623 (3)	0.51147 (9)	0.0463 (5)	
C36	0.23450 (10)	0.4594 (3)	0.52782 (8)	0.0434 (5)	
H36	0.2487	0.4193	0.5605	0.052*	
C37	0.35572 (11)	0.5171 (3)	0.51098 (10)	0.0534 (6)	
O31	0.37215 (8)	0.4790 (3)	0.55607 (7)	0.0824 (6)	
O32	0.39472 (9)	0.5595 (4)	0.47872 (8)	0.0914 (7)	
N33	0.16661 (14)	0.6480 (4)	0.38107 (9)	0.0734 (7)	
O33	0.20650 (13)	0.7306 (3)	0.35708 (8)	0.0999 (8)	
O34	0.10817 (12)	0.6161 (4)	0.36557 (8)	0.1032 (8)	
N35	0.11738 (10)	0.3995 (3)	0.54647 (9)	0.0627 (6)	
O35	0.05802 (9)	0.3917 (3)	0.53021 (9)	0.0903 (7)	
O36	0.13782 (10)	0.3596 (3)	0.58988 (8)	0.0875 (7)	

### 4-(4-Fluorophenyl)piperazin-1-ium 3,5-dinitrobenzoate (V) Atomic displacement parameters (Å^2^)

**Table:**

	*U*^11^	*U*^22^	*U*^33^	*U*^12^	*U*^13^	*U*^23^
N1	0.0360 (10)	0.109 (2)	0.0463 (12)	0.0062 (12)	−0.0019 (9)	0.0130 (13)
C2	0.0547 (15)	0.098 (2)	0.0529 (15)	−0.0039 (15)	−0.0078 (12)	−0.0124 (16)
C3	0.0596 (15)	0.0713 (17)	0.0491 (14)	0.0025 (13)	−0.0045 (11)	−0.0097 (13)
N4	0.0509 (11)	0.0569 (12)	0.0406 (10)	0.0022 (9)	−0.0044 (8)	−0.0004 (9)
C5	0.0530 (14)	0.0640 (16)	0.0548 (14)	0.0015 (12)	−0.0050 (11)	0.0042 (13)
C6	0.0490 (13)	0.0767 (18)	0.0541 (15)	0.0059 (13)	0.0015 (11)	0.0111 (14)
C21	0.0441 (12)	0.0557 (14)	0.0418 (12)	0.0004 (10)	0.0001 (10)	0.0037 (11)
C22	0.0615 (15)	0.0680 (17)	0.0505 (14)	0.0122 (13)	−0.0014 (12)	−0.0038 (13)
C23	0.0647 (16)	0.0760 (19)	0.0663 (18)	0.0187 (14)	−0.0085 (14)	0.0054 (15)
C24	0.0579 (15)	0.0756 (18)	0.0486 (15)	−0.0036 (14)	−0.0125 (12)	0.0146 (14)
F24	0.0917 (12)	0.1160 (14)	0.0683 (10)	0.0006 (10)	−0.0323 (9)	0.0193 (10)
C25	0.0721 (17)	0.0804 (19)	0.0411 (13)	−0.0046 (15)	−0.0028 (12)	−0.0039 (13)
C26	0.0602 (15)	0.0706 (17)	0.0474 (14)	0.0122 (13)	0.0006 (11)	−0.0045 (13)
C31	0.0402 (11)	0.0415 (12)	0.0455 (12)	0.0050 (9)	−0.0005 (10)	−0.0112 (10)
C32	0.0497 (13)	0.0487 (14)	0.0485 (13)	0.0029 (11)	0.0064 (10)	−0.0074 (11)
C33	0.0569 (14)	0.0505 (14)	0.0424 (12)	0.0085 (11)	−0.0053 (11)	−0.0033 (11)
C34	0.0440 (12)	0.0507 (14)	0.0595 (15)	0.0059 (11)	−0.0111 (11)	−0.0070 (12)
C35	0.0416 (11)	0.0429 (13)	0.0539 (13)	0.0013 (10)	0.0017 (10)	−0.0026 (11)
C36	0.0417 (11)	0.0429 (12)	0.0448 (12)	0.0050 (9)	−0.0020 (10)	−0.0039 (10)
C37	0.0401 (12)	0.0620 (16)	0.0579 (15)	0.0044 (11)	0.0029 (11)	−0.0119 (13)
O31	0.0419 (9)	0.1330 (19)	0.0699 (13)	0.0026 (11)	−0.0099 (9)	0.0154 (13)
O32	0.0466 (10)	0.152 (2)	0.0778 (13)	0.0040 (12)	0.0200 (10)	−0.0024 (14)
N33	0.0852 (18)	0.0794 (17)	0.0537 (14)	0.0249 (14)	−0.0067 (13)	0.0003 (13)
O33	0.1208 (19)	0.1093 (18)	0.0701 (14)	0.0175 (15)	0.0102 (13)	0.0309 (14)
O34	0.0853 (15)	0.146 (2)	0.0724 (14)	0.0255 (15)	−0.0304 (12)	0.0039 (14)
N35	0.0465 (12)	0.0626 (14)	0.0795 (16)	0.0019 (10)	0.0081 (11)	0.0074 (12)
O35	0.0412 (10)	0.1159 (18)	0.1140 (17)	−0.0061 (11)	0.0070 (10)	0.0177 (14)
O36	0.0689 (12)	0.1166 (18)	0.0783 (14)	−0.0009 (12)	0.0146 (11)	0.0302 (13)

### 4-(4-Fluorophenyl)piperazin-1-ium 3,5-dinitrobenzoate (V) Geometric parameters (Å, º)

**Table:**

N1—C6	1.481 (3)	C24—C25	1.358 (4)
N1—C2	1.482 (4)	C24—F24	1.366 (3)
N1—H11	0.94 (3)	C25—C26	1.380 (3)
N1—H12	0.95 (3)	C25—H25	0.9300
C2—C3	1.514 (3)	C26—H26	0.9300
C2—H2A	0.9700	C31—C32	1.386 (3)
C2—H2B	0.9700	C31—C36	1.386 (3)
C3—N4	1.453 (3)	C31—C37	1.515 (3)
C3—H3A	0.9700	C32—C33	1.373 (3)
C3—H3B	0.9700	C32—H32	0.9300
N4—C21	1.427 (3)	C33—C34	1.371 (3)
N4—C5	1.466 (3)	C33—N33	1.474 (3)
C5—C6	1.510 (3)	C34—C35	1.376 (3)
C5—H5A	0.9700	C34—H34	0.9300
C5—H5B	0.9700	C35—C36	1.383 (3)
C6—H6A	0.9700	C35—N35	1.470 (3)
C6—H6B	0.9700	C36—H36	0.9300
C21—C22	1.378 (3)	C37—O32	1.234 (3)
C21—C26	1.389 (3)	C37—O31	1.237 (3)
C22—C23	1.393 (3)	N33—O33	1.214 (3)
C22—H22	0.9300	N33—O34	1.223 (3)
C23—C24	1.356 (4)	N35—O36	1.217 (3)
C23—H23	0.9300	N35—O35	1.224 (2)
			
C6—N1—C2	111.42 (19)	C24—C23—C22	118.6 (2)
C6—N1—H11	107.0 (17)	C24—C23—H23	120.7
C2—N1—H11	107.1 (17)	C22—C23—H23	120.7
C6—N1—H12	109.2 (16)	C23—C24—C25	122.5 (2)
C2—N1—H12	109.2 (17)	C23—C24—F24	119.1 (3)
H11—N1—H12	113 (2)	C25—C24—F24	118.3 (2)
N1—C2—C3	111.3 (2)	C24—C25—C26	118.7 (2)
N1—C2—H2A	109.4	C24—C25—H25	120.7
C3—C2—H2A	109.4	C26—C25—H25	120.7
N1—C2—H2B	109.4	C25—C26—C21	121.1 (2)
C3—C2—H2B	109.4	C25—C26—H26	119.5
H2A—C2—H2B	108.0	C21—C26—H26	119.5
N4—C3—C2	109.5 (2)	C32—C31—C36	119.43 (19)
N4—C3—H3A	109.8	C32—C31—C37	120.0 (2)
C2—C3—H3A	109.8	C36—C31—C37	120.5 (2)
N4—C3—H3B	109.8	C33—C32—C31	119.7 (2)
C2—C3—H3B	109.8	C33—C32—H32	120.2
H3A—C3—H3B	108.2	C31—C32—H32	120.2
C21—N4—C3	116.93 (19)	C34—C33—C32	122.5 (2)
C21—N4—C5	114.89 (18)	C34—C33—N33	118.2 (2)
C3—N4—C5	109.80 (19)	C32—C33—N33	119.3 (2)
N4—C5—C6	110.1 (2)	C33—C34—C35	116.9 (2)
N4—C5—H5A	109.6	C33—C34—H34	121.5
C6—C5—H5A	109.6	C35—C34—H34	121.5
N4—C5—H5B	109.6	C34—C35—C36	122.8 (2)
C6—C5—H5B	109.6	C34—C35—N35	118.4 (2)
H5A—C5—H5B	108.1	C36—C35—N35	118.8 (2)
N1—C6—C5	110.9 (2)	C35—C36—C31	118.7 (2)
N1—C6—H6A	109.5	C35—C36—H36	120.6
C5—C6—H6A	109.5	C31—C36—H36	120.6
N1—C6—H6B	109.5	O32—C37—O31	125.7 (2)
C5—C6—H6B	109.5	O32—C37—C31	117.4 (2)
H6A—C6—H6B	108.1	O31—C37—C31	116.8 (2)
C22—C21—C26	118.3 (2)	O33—N33—O34	124.4 (3)
C22—C21—N4	123.3 (2)	O33—N33—C33	117.9 (3)
C26—C21—N4	118.3 (2)	O34—N33—C33	117.7 (3)
C21—C22—C23	120.8 (2)	O36—N35—O35	123.9 (2)
C21—C22—H22	119.6	O36—N35—C35	118.3 (2)
C23—C22—H22	119.6	O35—N35—C35	117.8 (2)
			
C6—N1—C2—C3	−53.0 (3)	C37—C31—C32—C33	−179.1 (2)
N1—C2—C3—N4	57.3 (3)	C31—C32—C33—C34	−0.6 (4)
C2—C3—N4—C21	165.3 (2)	C31—C32—C33—N33	178.7 (2)
C2—C3—N4—C5	−61.5 (3)	C32—C33—C34—C35	0.3 (4)
C21—N4—C5—C6	−164.08 (19)	N33—C33—C34—C35	−179.0 (2)
C3—N4—C5—C6	61.7 (3)	C33—C34—C35—C36	0.4 (3)
C2—N1—C6—C5	52.6 (3)	C33—C34—C35—N35	−179.9 (2)
N4—C5—C6—N1	−56.8 (3)	C34—C35—C36—C31	−0.8 (3)
C3—N4—C21—C22	10.9 (3)	N35—C35—C36—C31	179.5 (2)
C5—N4—C21—C22	−120.0 (3)	C32—C31—C36—C35	0.5 (3)
C3—N4—C21—C26	−167.2 (2)	C37—C31—C36—C35	179.8 (2)
C5—N4—C21—C26	61.9 (3)	C32—C31—C37—O32	−5.1 (3)
C26—C21—C22—C23	0.8 (4)	C36—C31—C37—O32	175.6 (2)
N4—C21—C22—C23	−177.3 (2)	C32—C31—C37—O31	173.0 (2)
C21—C22—C23—C24	−1.3 (4)	C36—C31—C37—O31	−6.3 (3)
C22—C23—C24—C25	0.8 (4)	C34—C33—N33—O33	166.0 (2)
C22—C23—C24—F24	−179.6 (2)	C32—C33—N33—O33	−13.4 (4)
C23—C24—C25—C26	0.0 (4)	C34—C33—N33—O34	−14.6 (3)
F24—C24—C25—C26	−179.5 (2)	C32—C33—N33—O34	166.0 (2)
C24—C25—C26—C21	−0.5 (4)	C34—C35—N35—O36	−174.5 (2)
C22—C21—C26—C25	0.0 (4)	C36—C35—N35—O36	5.2 (3)
N4—C21—C26—C25	178.2 (2)	C34—C35—N35—O35	5.1 (3)
C36—C31—C32—C33	0.2 (3)	C36—C35—N35—O35	−175.2 (2)

### 4-(4-Fluorophenyl)piperazin-1-ium 3,5-dinitrobenzoate (V) Hydrogen-bond geometry (Å, º)

**Table:**

*D*—H···*A*	*D*—H	H···*A*	*D*···*A*	*D*—H···*A*
N1—H11···O31	0.94 (3)	1.77 (3)	2.681 (3)	164 (3)
N1—H12···O32^i^	0.95 (3)	1.80 (3)	2.731 (3)	165 (3)
C23—H23···*Cg*3^ii^	0.93	2.88	3.787 (3)	167

Symmetry codes: (i) −*x*+1, −*y*+1, −*z*+1; (ii) −*x*+3/2, *y*−1/2, −*z*+3/2.
